# Bioconversion of Biologically Active Indole Derivatives with Indole-3-Acetic Acid-Degrading Enzymes from *Caballeronia glathei* DSM50014

**DOI:** 10.3390/biom10040663

**Published:** 2020-04-24

**Authors:** Mikas Sadauskas, Roberta Statkevičiūtė, Justas Vaitekūnas, Rolandas Meškys

**Affiliations:** Department of Molecular Microbiology and Biotechnology, Institute of Biochemistry, Life Sciences Center, Vilnius University, Saulėtekio al. 7, LT-10257 Vilnius, Lithuania; roberta.statkeviciute@gmc.stud.vu.lt (R.S.); justas.vaitekunas@bchi.vu.lt (J.V.); rolandas.meskys@bchi.vu.lt (R.M.)

**Keywords:** indole-3-acetic acid, indole-3-propionic acid, indole-3-butyric acid, biodegradation, *Caballeronia glathei*, bioconversion

## Abstract

A plant auxin hormone indole-3-acetic acid (IAA) can be assimilated by bacteria as an energy and carbon source, although no degradation has been reported for indole-3-propionic acid and indole-3-butyric acid. While significant efforts have been made to decipher the Iac (indole-3-acetic acid catabolism)-mediated IAA degradation pathway, a lot of questions remain regarding the mechanisms of individual reactions, involvement of specific Iac proteins, and the overall reaction scheme. This work was aimed at providing new experimental evidence regarding the biodegradation of IAA and its derivatives. Here, it was shown that *Caballeronia glathei* strain DSM50014 possesses a full *iac* gene cluster and is able to use IAA as a sole source of carbon and energy. Next, IacE was shown to be responsible for the conversion of 2-oxoindole-3-acetic acid (Ox-IAA) intermediate into the central intermediate 3-hydroxy-2-oxindole-3-acetic acid (DOAA) without the requirement for IacB. During this reaction, the oxygen atom incorporated into Ox-IAA was derived from water. Finally, IacA and IacE were shown to convert a wide range of indole derivatives, including indole-3-propionic acid and indole-3-butyric acid, into corresponding DOAA homologs. This work provides novel insights into Iac-mediated IAA degradation and demonstrates the versatility and substrate scope of IacA and IacE enzymes.

## 1. Introduction

**Figure 1:**
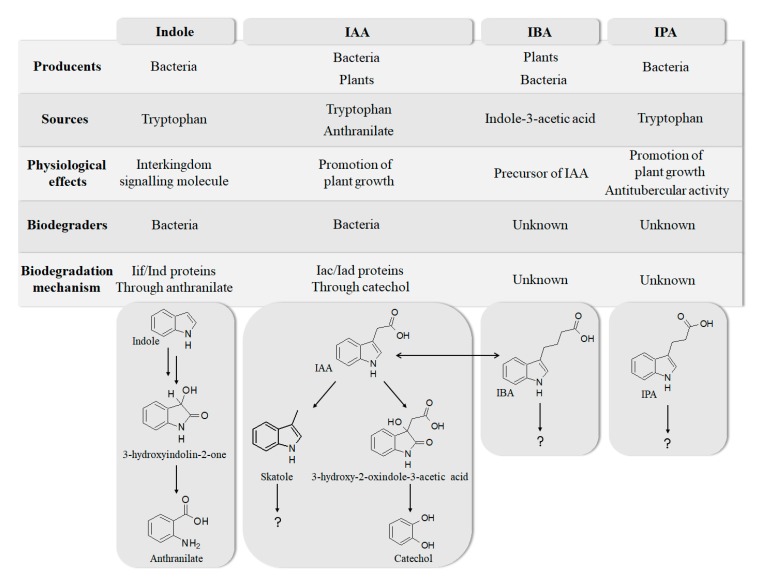
Metabolism of biologically relevant derivatives of indole.

Indole and its derivatives comprise a group of biologically active *N*-heterocyclic compounds. Indole itself has recently been recognized as an interkingdom signaling molecule [1]. It is produced mainly by gut bacteria following the activity of tryptophanase [2], but can alter the physiology and metabolism of a very wide range of organisms, including the producent itself [3,4], other bacteria [5,6], eukaryotes [7], and even mammals [8,9]. The 3-substituted derivatives of indole bearing a carboxylic acid group are regarded as auxins, the plant growth-regulating hormones. These molecules can promote plant growth at basically all levels, including molecular, cellular, tissue, organ and whole plant levels [10]. The effect of auxins is concentration-dependent, requiring a strict regulation of auxin synthesis, degradation, conjugation, and import/export [11]. While indole-3-acetic acid (IAA or heteroauxin) is regarded as the most potent auxin with the strongest plant growth-regulating effects, other auxins that share similar structural scaffold were characterized as well, including indole-3-propionic acid (IPA) and indole-3-butyric acid (IBA) [12]. Surprisingly, the physiological effects of IPA, a product of bacterial tryptophan deamination, has recently been described not only in plants, but in the mammalian organisms as well (Figure 1). Increased concentration of IPA has been linked to lower risk of type 2 diabetes in humans [13,14]. Moreover, IPA has been reported to provide beneficial effects for liver functions [15], perform as a biomarker for development of chronic kidney disease [16], and help to reduce the body weight in antibiotic-treated mice [17]. In addition, the activity of IPA against *M. tuberculosis* has been demonstrated [18] and later attributed to the suppression of tryptophan biosynthesis through the inhibition of anthranilate synthetase TrpE [19].

The aforementioned derivatives of indole-3-carboxylic acids can be present at large concentrations in bacteria-dominated niches, such as soil and gut, thus it would make sense for bacteria to use these compounds as carbon or energy sources. Indeed, bacterial degradation of indole and IAA has long been recognized [20,21]. Meanwhile no processes of biodegradation for IPA nor IBA has been reported so far (Figure 1). The bacterial degradation of IAA has been attributed to the activity of Iac proteins (indole acetic acid), encoded by *iac* gene cluster. Four bacterial strains with demonstrated aerobic IAA degradation capability and known genetic determinants for biodegradation–*Pseudomonas putida* 1290 [21], *Acinetobacter baumannii* ATCC19606 [22], *Paraburkholderia phytofirmans* PsJN [23], and *Enterobacter soli* LF7 [24]—were shown to follow the Iac-mediated biodegradation pathway. However, the information about biological cycle of IPA and IBA in different eco-niches is scarce. Numerous plants are able to convert the auxin precursor IBA into active IAA [25,26]. In particular, it has been suggested that, in *Arabidopsis,* a fatty acid beta-oxidation takes place in this process [27]. The fate of IPA is even less understood.

In the proposed mechanism of Iac-based biodegradation, IacA acts as an initial IAA oxygenase, producing 2-oxoindole-3-acetic acid (Ox-IAA) [22,28,29], which is then transformed to 3-hydroxy-2-oxindole-3-acetic acid (DOAA) by IacE, possibly involving IacB [23] as well. The end-product of Iac-mediated degradation is catechol [28], which is further oxidized by a catechol dioxygenase, the genes of which (*catABC*) are located in close proximity to the *iac* cluster [23,24,28]. Still, several questions remain unanswered in Iac-based biodegradation of IAA. While the end product of initial oxidation of IAA is presumed to be Ox-IAA, the most stable and easily identifiable end product of indole oxidation reaction with IifC/IndA indole monooxygenases was indigo. The intermediate reaction product of IifC/IndA monooxygenases has long been elusive until a recent demonstration that indole-2,3-epoxide is the unstable intermediate [30]. Hence, the reaction product of IacA-catalyzed reaction and the reaction mechanism remain inconclusive. Also, IacE has been suggested to introduce another oxygen atom into Ox-IAA, therefore acting as an oxygenase. However, the amino acid sequence of IacE showed the highest sequence identity to short-chain dehydrogenases/reductases, which usually act as reductases [31] performing the NAD(P)(H)-dependent oxidoreduction of hydroxy/keto groups and usually does not incorporate oxygen. Finally, no intermediate has been proposed which could appear during the conversion of DOAA into catechol. Recently, an enzymatic decarboxylation of IAA to skatole by indoleacetate decarboxylase (Iad) has been identified [32] adding a new metabolic pathway to the IAA catabolism. 

**Figure 2:**
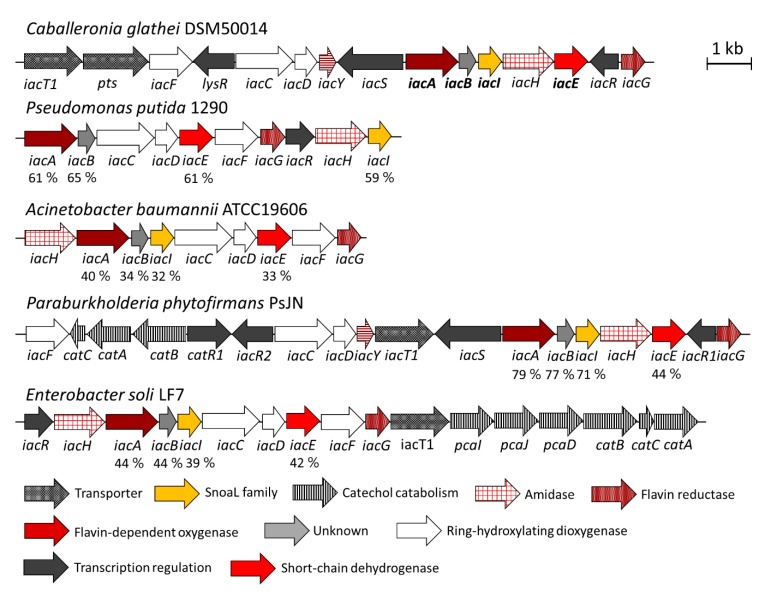
Distribution of *iac* and related genes in the genomes of known IAA-degrading microorganisms and *C. glathei* DSM50014. The genes of the enzymes that were studied in this work are highlighted in bold. Accession numbers of the proteins studied in this work are: IacA–WP_035925671, IacB–WP_035925668, IacI–WP_035925861, IacE–WP_035925663. The identity percentage between homologous genes that were studied in this report are also indicated.

*Caballeronia glathei*, isolated as *Pseudomonas glathei* [33], later reclassified as *Burkholderia glathei* [34] and *Paraburkholderia glathei* [35], and finally as *C. glathei* [36], is a gram-negative bacterium and belongs to the class of β-Proteobacteria. *C. glathei* can inhabit different environments but is mainly found in soil. Although most Burkholderia-related microorganisms are relatively well-studied because of plant growth-promoting characteristics, biocontrol of plant diseases, or even being opportunistic pathogens for plants and humans [37], little is known about the ecological role of *C. glathei*. It has been demonstrated that *C. glathei* can establish a close relationship with soil-dominating fungi, which provides the bacterium with additional ecological fitness [38]. A recently published genome sequence of the type strain DSM50014 (GenBank RefSeq no. NZ_JFHC00000000.1) [39] contains a full set of iac genes, providing a framework for studying the functions of Iac proteins. Compared to other IAA-degrading strains that are publicly available, the iac locus in C. glathei DSM50014 is less interrupted by other genes (Figure 2) and comprises all iac genes that are known to date: iacA–I and iacR, iacS, iacT1, iacY. Therefore, the goal of this study was to analyze the initial steps of the catabolism of IAA in *C. glathei* and to offer additional data that would help to elucidate the Iac-mediated pathway of IAA biodegradation in detail. The second objective was to characterize a substrate scope (a range of converted/unconverted substrates by the enzyme without specifying the substrate preference, which would require enzyme kinetics) of the IacA and IacE proteins.

## 2. Materials and Methods 

### 2.1. Reagents, Bacterial Strains and Growth Conditions

All IAA derivatives used in this study (indole-3-acetic acid, 3-(2-hydroxyethyl)indole, ethyl-3-indole-acetate, 3-(3-hydroxypropyl)indole, 3-indoleacetonitrile, indole-3-acetamide, indole-3-butyric acid, DL-indole-3-lactic acid, indole-3-propionic acid, tryptamine, indole-3-acrycil acid and indole-3-carboxylic acid) and H_2_^18^O were purchased from Sigma-Aldrich. All cloning and protein expression reagents were from ThermoFisher Scientific (Vilnius, Lithuania). All other reagents used in this study were of analytical or higher grade.

*Caballeronia glathei* strain DSM50014 was obtained from the German Collection of Microorganisms and Cell Cultures (DSMZ), Braunschweig, Germany. This strain was routinely cultivated in M1 medium (5 g L^−1^ peptone, 3 g L^−1^ meat extract, pH 7). For the IAA assimilation experiments, *C. glathei* DSM50014 was cultivated in M9 medium (3.5 g L^−1^ Na_2_HPO_4_, 1.5 g L^−1^ KH_2_PO_4_, 2.5 g L^−1^ NaCl, 0.2 g L^−1^ MgSO_4_, 0.01 g L^−1^ CaCl_2_). *Escherichia coli* strains used in this study are listed in Supplementary Table S2. All *E. coli* strains were cultivated in LB medium. Ampicillin (50 μg mL^−1^) and streptomycin (30 μg mL^−1^) were added when necessary.

### 2.2. Cloning and Expression of Iac Genes

Genomic DNA from *C. glathei* was extracted as described [40]. *iacA, iacE* and *iacB* genes were amplified from genomic DNA of *C. glathei* DSM50014 with oligonucleotides listed in Supplementary Table S1. Cloning and expression of *iacA, iacB,* and *iacE* genes was performed following the general protocol described previously [41], except that the expression of IacE was induced with 0.1 mM IPTG and performed overnight at 16 °C. For protein co-expression, *iacA* was cloned to pET-28c(+) to obtain pET28-iacA. *iacB* and *iacE* were cloned to the MCS1 and MCS2, respectively, of pCDFDuet-1 to obtain pCDFDuet-iacB and pCDFDuet-iacE.

### 2.3. Whole-Cell Bioconversion

*E. coli* cells producing recombinant IacA, IacB and IacE proteins or their combinations were suspended in potassium phosphate buffer (10 mM, pH 7.7) supplemented with succinate (5 mM) to reach the 2× concentration of an initial culture and the IAA or derivative of IAA was added to a final concentration of 2 mM. Incubation of whole cells was performed with agitation (180 RPM, Innova44 Shaker, Eppendorf) at 30 °C overnight. Cells were removed by centrifugation at 16,000× *g* for 5 min and the supernatant was subjected to HPLS/MS analysis.

### 2.4. Analytical Techniques

Substrate consumption and formation of products during the bioconversion experiments was analyzed spectrophotometrically by PowerWave XS plate reader (BioTek Instruments, Inc, Winooski, VT, USA) or the samples were mixed with an equal volume of acetonitrile and subjected to HPLC/MS analysis, which was performed as described [41]. Bioconversion efficiency of IacA was calculated as described [42], except that absorbance area at 280 nm was used rather than 254 nm. Three independent experiments were performed for each substrate and bioconversion efficiency of IacA is presented as mean ± SD.

IacE was purified by Ni-NTA affinity chromatography through C-terminal 6xHisTag as described in [41]. Enzymatic activity of purified IacE was monitored in reaction mixtures containing purified Ox-IAA, 1 mM of different cofactors (NAD^+,^ NADP^+^, NADH or NADPH) and 1 μg of purified IacE. Reactions were incubated at 30 °C for different time intervals and analyzed with HPLC/MS as described above.

Purification of Ox-IAA and DOAA was performed by using reversed-phase preparative fast performance liquid chromatography essentially as described earlier [43]. Subsequently, ^13^C and ^1^H NMR spectra of purified DOAA were recorded as described [42].

2-(3-hydroxy-2-oxoindolin-3-yl)acetic acid (DOAA). White solid. ^1^H NMR (400 MHz, DMSO-d6): δ = 1.89 (d, *J* = 14.9 Hz, 1H, CH), 2.28 (d, *J* = 14.9 Hz, 1H, CH), 6.76 (d, *J* = 7.6, 1H, CH), 6.89 (t, *J* = 7.5, 1H, CH), 7.14 (t, 1H, CH), 7.32 (d, *J* = 7.4, 1H, CH) 9.72 (s, 1H, CH) 10.12 (s, 1H, NH). ^13^C NMR (100 MHz, DMSO-d6): δ = 42.5, 74.3, 109.7, 121.8, 124.3, 128.7, 134.8, 141.4, 174.2, 179.3.

### 2.5. Utilization of H_2_^18^O

Following an overnight induction of expression or co-expression of genes *iacA*, *iacB* and *iacE* in *E. coli*, cells were washed with potassium phosphate buffer and suspended in H_2_^18^O, containing 2 mM of IAA. For consumption of Ox-IAA, cells were suspended in H_2_^18^O mixed with the solution of Ox-IAA (ratio 1:1). Bioconversion in H_2_^18^O and analysis of reaction products was performed as described above.

## 3. Results

### 3.1. Identification of Caballeronia Glathei DSM50014 as a Biodegrader of IAA

*Caballeronia glathei* DSM50014 was obtained from the DSMZ collection bank and was identified to possess a full set of *iac* genes (sequence accession number NZ_JFHC01000015.1, nucleotide positions 99054–113486, Figure 2). First, this strain was tested for the capability to assimilate IAA. The growth was visible on M9 minimal medium agar plates supplemented with 1 mM IAA after the incubation period of five days (Supplementary Figure S1) confirming the ability of *C. glathei* DSM50014 to use IAA as a sole carbon and energy source.

**Figure 3:**
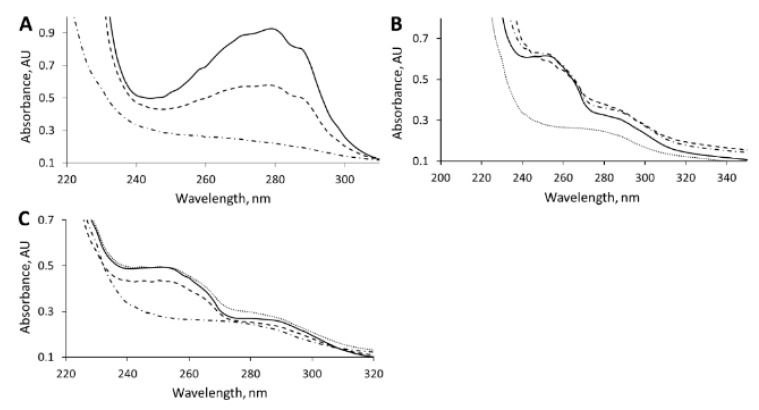
The whole-cell bioconversion of indole-3-acetic acid, 1 mM (**A**), 3-hydroxyindolin-2-one (**B**) and 3-hydroxy-2-oxindole-3-acetic acid (DOAA) (**C**). Bioconversion was performed at 30 °C overnight. Solid line–negative control (no cells), dashed line–*C. glathei* DSM50014, dash-dotted line–IAA (indole-3-acetic acid)-induced *C. glathei* DSM50014, dotted line–indole-induced *Acinetobacter* sp. O153.

In order to test whether the assimilation of IAA occurred through Iac-mediated pathway, whole cells of *C. glathei* were tested for the ability to consume DOAA, an intermediate in the Iac-mediated degradation pathway. IAA-induced *C. glathei* cells consumed both IAA and DOAA at the faster rate compared to the uninduced cells (Figure 3A,B), suggesting that both IAA degradation in *C. glathei* DSM50014 was an inducible process and DOAA was an intermediate compound during the assimilation. On the other hand, the DOAA counterpart in the indole degradation process, 3-hydroxyindolin-2-one, was not consumed by *C. glathei* DSM50014 as opposed to a natural indole-degrader *Acinetobacter* sp. strain O153 (Figure 3C). The absence of absorbance spectra that could indicate possible conversion products showed that *C. glathei* cells fully assimilated IAA and DOAA. Taken together, these results confirmed that *C. glathei* strain DSM50014 followed an Iac-mediated degradation of IAA.

### 3.2. IacA- and IacE-Catalyzed Reactions in IAA Degradation

**Figure 4:**
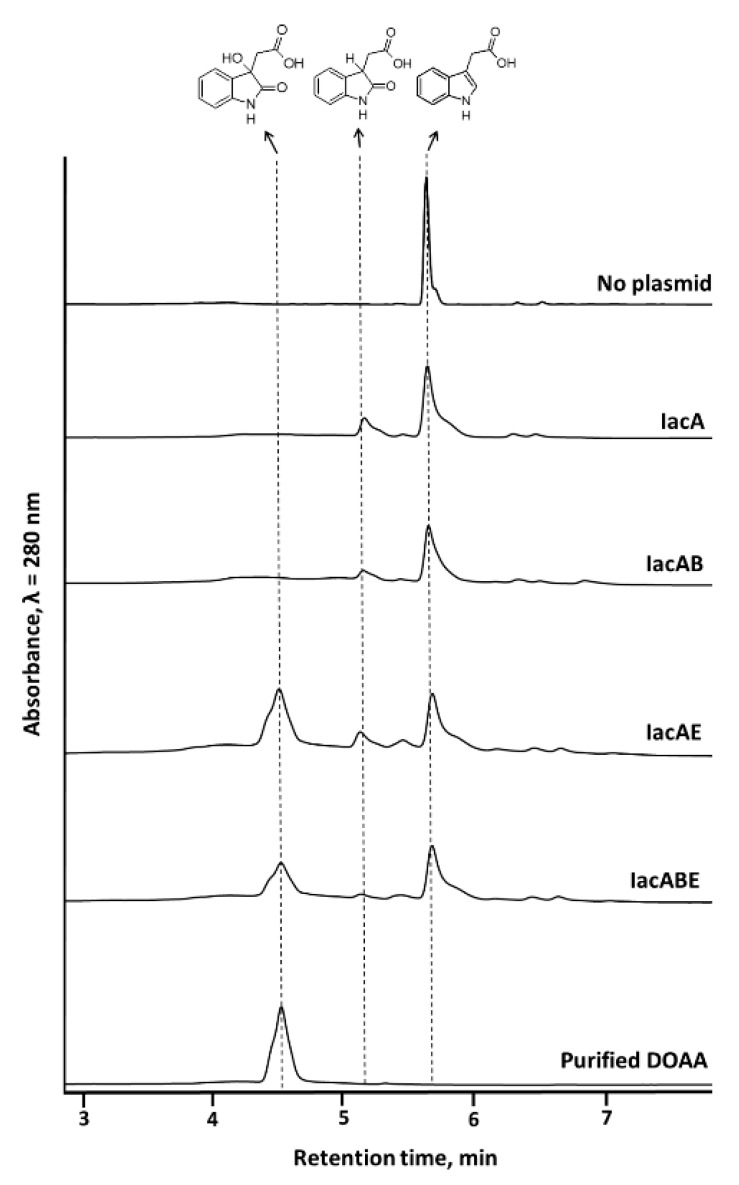
HPLC analysis of the bioconversion reaction mixture of IAA (1 mM) with *E. coli* whole-cells expressing different combination of IacA, IacB and IacE proteins. Formulas of the identified compounds are presented. Bioconversion reactions were carried out at 30 °C overnight.

To clarify the roles of individual Iac proteins, which were presumed to convert IAA into DOAA, *iacA, iacE,* and *iacB* genes were cloned to compatible plasmids and expressed in *E. coli* to obtain a bioconversion platform with different Iac protein combinations. IacA was annotated as a flavin-dependent acyl-CoA dehydrogenase (ACAD) family protein, IacE as a short-chain dehydrogenase/reductase, and no function prediction could be obtained from the sequence of IacB [23]. IacG was annotated as a flavin reductase and should provide a reduced flavin for the initial oxidation of IAA. However, as *E. coli* possesses numerous flavin reductases, which were shown to complement similar heterologous bioconversion reactions [41], IacG was omitted from the construction of IAA-converting *E. coli* strain. IacA has been demonstrated to oxidize IAA, but the exact reaction mechanism has not been elucidated, possibly due to instability of reaction intermediates as has been the case with biological indole oxidation [30]. Thus, the attention was focused on testing whether Ox-IAA was an intermediate compound in IAA biodegradation, and if so, which enzymes were required for the conversion of Ox-IAA into DOAA. Bioconversion of IAA by using *E. coli* cells expressing IacAE proteins resulted in accumulation of three compounds (Figure 4). The first compound with retention time of 5.2 min and molecular mass of 191 Da was also the major product during the conversion of IAA by IacA, assigning this peak as Ox-IAA. In both these reactions, a second product was also observed with a retention time of 5.5 min and molecular mass of 382 (absorbance maxima at 241 nm and 293 nm), which could be a dimer of Ox-IAA, but the exact structure of this compound remained unidentified. The third compound with retention time of 4.6 min and molecular mass of 207 Da was only observed during the conversion of IAA by IacAE. It also possessed an absorbance spectrum resembling that of 3-hydroxyindolin-2-one, an intermediate compound (absorbance maxima at 254 nm and 291 nm) in indole biodegradation [41]. The latter product of IAA bioconversion was purified by using RP-FPLC. ^1^H and ^13^C NMR spectra (Supplementary Figures S19 and S20) corresponded to the ones published earlier [23], confirming that this compound was DOAA. *E. coli* with the combination of IacAB proteins did not produce DOAA, and reaction products profile was almost identical to that when using single IacA protein (Ox-IAA and the unidentified compound with 5.5 min retention time), except for a small peak at 6.9 min, which could not be identified. Also, no qualitative differences were observed between the bioconversion products of IacAE- and IacABE-carrying *E. coli* cells. The concentration of DOAA differed in these reactions, possibly because *E. coli* had to synthesize different sets of proteins. All these results confirmed that IacE catalyzed the second reaction after initial IAA oxygenation by IacA to obtain DOAA in *E. coli* system without the requirement of IacB.

**Figure 5:**
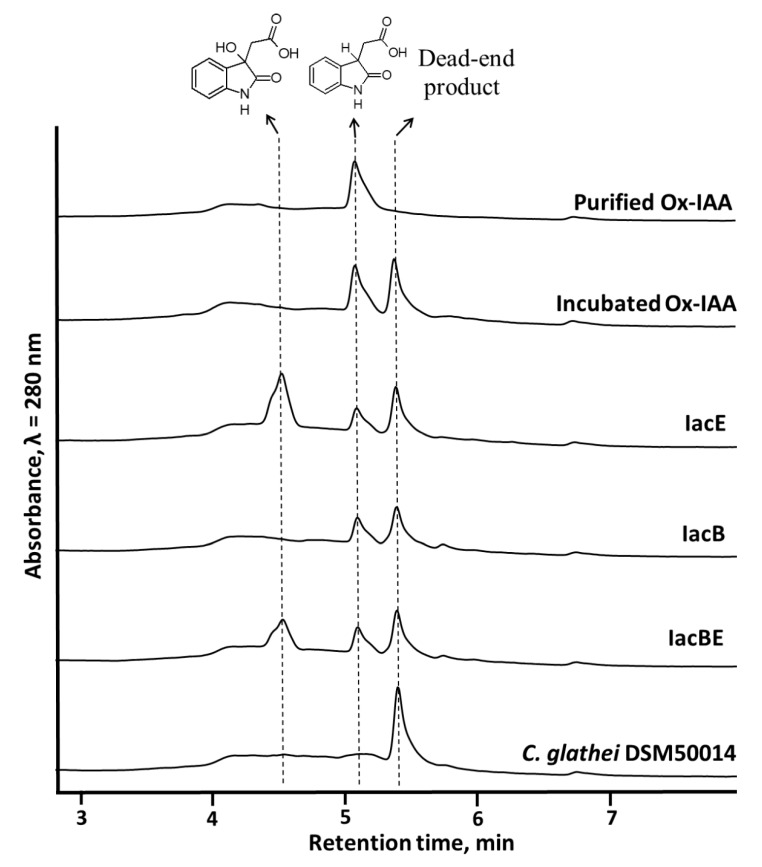
HPLC analysis of the bioconversion reaction mixture of 2-hydroxyindole-3-acetic acid (Ox-IAA) with *E. coli* cells expressing different combination of IacB and IacE proteins or *C. glathei* DSM50014. Bioconversion reactions were carried out at 30 °C overnight.

The product of IacA-catalyzed IAA conversion, identified as Ox-IAA (retention time 5.3 min, molecular mass 191, absorbance maxima 241 nm and 293 nm) was also purified and tested as a substrate for both IacE- and IacB-expressing *E. coli*. Firstly, Ox-IAA was found to be unstable as it formed the product with unidentified structure as described above (retention time 5.5 min, molecular mass 382). In spite of that, Ox-IAA was converted to DOAA only in the presence of IacE (Figure 5) and the addition of IacB did not show any changes in the profile of the reaction of products. In addition, IAA-induced *C. glathei* DSM50014 cells were able to consume Ox-IAA completely (Figure 5), but did not consume the product with retention time 5.5 min and unknown structure, strongly suggesting that the latter compound can be a dead-end product. These results further supported the notion that IacB was not involved in the production of DOAA, and suggested that Ox-IAA was an intermediate compound in IAA biodegradation rather than a dead-end product.

### 3.3. Substrate Scope of IacA and IacE Proteins

To test the substrate scope of IacA and IacE, different derivatives of indole harboring different groups at the third position were tested as substrates with *E. coli* cells co-expressing IacA and IacE. Among 11 tested derivatives, tryptamine, indole-3-acrycil acid and indole-3-carboxylic acid were not oxidized by IacA. The rest of the compounds such as 3-(2-hydroxyethyl)indole, ethyl-3-indole-acetate, 3-(3-hydroxypropyl)indole, 3-indoleacetonitrile, indole-3-acetamide, indole-3-butyric acid, DL-indole-3-lactic acid and indole-3-propionic acid were oxidized to corresponding 2-oxo derivatives, albeit with different efficiency (Table 1, Supplementary Figure S5–S16). The two factors that govern the substrate scope of IacA could be the length of the carbon atom side chain, since the only substrate with less than two carbon atoms in the side chain (indole-3-carboxylic acid) was not oxidized, and the presence of the double bond in the side chain (indole-3-acrylic acid). Next, IacE was found to be capable of converting 3-(2-hydroxyethyl)indol-2-one, ethyl-(2-oxo-indol-3-yl)acetate, 3-(3-hydroxypropyl)indol-2-one, 2-oxo-3-indoleacetonitrile, 2-oxindole-3-butyric acid and 2-oxindole-3-propionic acid into corresponding DOAA homologs. Collectively, these results demonstrate a wide substrate scope of both IacA and IacE proteins.

### 3.4. Oxygen Incorporated into DOAA is Derived from Water

**Figure 6:**
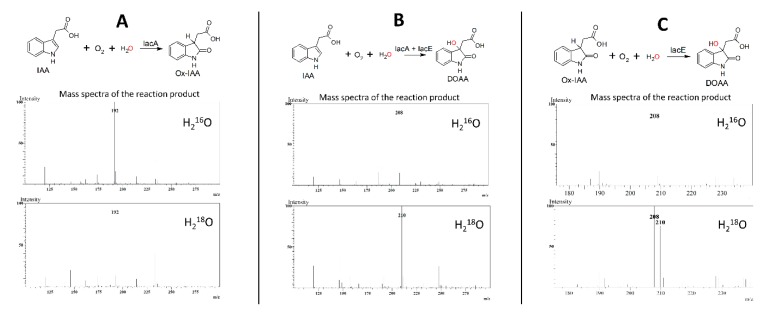
Mass spectra of Ox-IAA and DOAA obtained by using *E. coli* cells expressing IacA and IacE in H_2_^16^O environment and H_2_^18^O environment. (**A**)—Ox-IAA from IAA + IacA, (**B**)—DOAA from IAA + IacAE, (**C**)—DOAA from Ox-IAA + IacE. All mass spectra that are presented were recorded in positive ionization mode.

To gain insight into the reaction mechanism of IacAE-catalyzed IAA conversion into DOAA, H_2_^18^O was used to trace the origin of oxygen atoms incorporated during IAA oxygenation. The molecular mass of Ox-IAA remained unchanged when *E. coli* cells expressing IacA were used for bioconversion of IAA in H_2_^18^O environment (Figure 6A). However, DOAA with molecular mass of 209 was clearly observed in two cases: when *E. coli* cells expressing IacAE were used for bioconversion of IAA in H_2_^18^O environment and with Ox-IAA and IacE-expressing *E. coli* cells in H_2_^18^O environment (Figure 6B). Suspension of *E. coli* cells in H_2_^18^O and solution of Ox-IAA in H_2_^16^O were mixed in a volume ration of 1:1 for the experiments described in Figure 6C, explaining the presence of [M+H]^+^ ions 208 and 210 with comparable intensities. Taken together, these findings demonstrated that the oxygen atom at C3 in DOAA had originated from water.

## 4. Discussion

### 4.1. Involvement and Role of IacB in IAA Biodegradation

Recently, *Cupriavidus pinatubonensis* JMP134 cells carrying recombinant IacB, IacE or IacBE proteins were tested for ability to consume the products of IAA conversion by IacA (most likely Ox-IAA), and strains carrying IacE and IacBE were confirmed to be able to produce DOAA, with the latter combination producing higher amounts of DOAA [23]. This led to the hypothesis that IacB might be an auxiliary or accessory protein for IacE [23]. In this report, similar amounts of DOAA were produced with IacE- and IacBE-expressing *E. coli* cells using Ox-IAA as substrate. Also, no DOAA production was observed with cells expressing IacB only, which was in agreement with the activity of IacB expressed in *C. pinatubonensis* JMP134. A conversion of Ox-IAA into DOAA with cells expressing IacE also dismissed the proposed second attack of IacA [23]. Also, bioconversion of IAA homologs into corresponding DOAA-like derivatives was achieved with IacAE proteins only, strongly suggesting that IacB does not participate in the first two reactions of IAA degradation. On the other hand, an endogenous protein with a similar function might be present in *E. coli* or *C. pinatubonensis*, complementing the absence of IacB in IacE-expressing cells. No proteins with significant sequence similarity to IacB could be detected in the genomes of *E. coli* and *C. pinatubonensis* by using blastp tool [44], and since no function can be deduced from the sequence of this protein, the role of IacB in IAA degradation remains unclear.

### 4.2. The Role of Other Iac Proteins and Analogies with Indole Biodegradation

**Figure 7:**
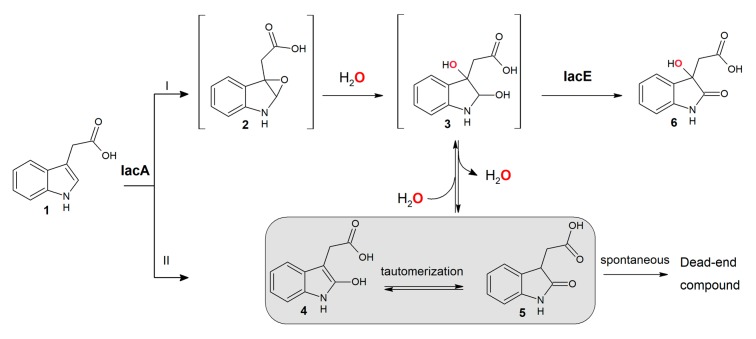
Proposed pathways of IacAE-mediated conversion of IAA into DOAA. 1—IAA, 2—2,3-epoxyIAA, 3—2,3-dihydroxyindoline-3-acetic acid, 4—2-hydroxyIAA, 5—Ox-IAA, 6—DOAA. ^18^O atom is marked in red.

A recently described mechanism for aerobic indole degradation [41,45] shows some similarities with an aerobic degradation of IAA. First of all, the two molecules share the same scaffold and also perform functions as signaling molecules, albeit in different organisms [1,10]. Most microorganisms with reported indole or IAA degradation capability belong to Proteobacteria, namely *Pseudomonas, Acinetobacter*, *Burkholderia* and related genera. The composition of genes involved in indole and IAA degradation (*iif* and *iac*, respectively) and their functions also appear to be similar. Both IifC and IacA are flavin-dependent oxygenases requiring a flavin reductase (IifD and IacG, respectively). However, these enzymes belong to different classes of flavin-dependent oxygenases: indole monooxygenase IifC is a member of the group E monooxygenases [30] while IacA possesses an ACAD fold and belongs to the group D of flavin monooxygenases [46]. Remarkably, IacA enzymes from IAA-degrading organisms form a separate branch from other group D flavin-dependent oxygenases (Figure S22) and could possibly represent a new group of epoxidation-catalyzing enzymes in this group. It has been proposed that IifC converts indole to an unstable epoxide. Then, indole-2,3-epoxide rapidly hydrolyzes to a diol, which is a substrate for the IifB dehydrogenase [27,41]. In the absence of the dehydrogenase, the epoxide spontaneously loses the water molecule, and the formed 3-indoxyl dimerizes into indigo [41]. However, in the case of IAA and IacA, a mechanism of oxidation of the IAA is not clear. Based on experiments using H_2_^18^O, it can be proposed that: i) IacA forms epoxide (Figure 7, reaction I), the latter in a non-enzymatic acid-catalyzed process, due to which the nucleophile attacks the more substituted carbon because it is this carbon that holds a greater degree of positive charge, is hydrolyzed to a diol, which, after spontaneous dehydration, forms Ox-IAA (in the case of a basic epoxide ring opening Ox-IAA would contain ^18^O originated from water, since the reaction occurs by an S_N_2 mechanism, and the less substituted carbon is the site of nucleophilic attack); ii) the primary product of IacA-catalyzed reaction is 2-hydroxy-IAA (Figure 7, reaction II), which tautomerizes into Ox-IAA. Also, it should be stressed that it would be impossible to distinguish between the two pathways if ^18^O_2_ is applied instead of atmospheric oxygen.

Comparing to catabolism of indole, a more complicated situation is observed at the next step of IAA degradation. The second reaction of indole and IAA biodegradation is catalyzed by a short chain dehydrogenase/reductase IifB and IacE, respectively. Since IifB performs the oxidation of 2,3-dihydroxyindoline during indole biodegradation [30,41], an analogous function might be hypothesized for IacE as well. In such a case, IacA produces an epoxide, similarly to IifC [30], then, after spontaneous hydrolysis, IacE oxidizes a hydroxy group at C2 position of the formed indoline derivative to yield DOAA (Figure 7). Hence, the observed Ox-IAA should be a dead-end product of the IacA-catalyzed reaction (as 3-indoxyl/indigo in the case of IifC). However, Ox-IAA was fully consumed by IAA-induced *C. glathei* cells as well as by *E. coli* cells carrying IacE protein or by a cell-free extract prepared from *E. coli* cells producing IacE protein and supplemented with NADH. However, all attempts to register a conversion of Ox-IAA in the presence of NAD(P)^+^ or NAD(P)H by the purified recombinant IacE protein have been unsuccessful. In addition, a spontaneous transformation of Ox-IAA to the unidentified product (Figure 5, retention time 5.4 min) is observed even in the absence of any enzymes. A similar process takes place with all Ox-IAA homologs containing a carboxylic group (i.e., 2-hydroxyindole-3-propionic acid and 2-hydroxyindole-3-butyric acid), suggesting an instability of such compounds under experimental conditions. However, the most remarkable feature of the IacE-mediated reaction is that the oxygen atom from water has been introduced into the product—DOAA (Figure 6).

IacE possesses a Rossman fold domain, strongly indicating a redox function of this enzyme. Also, according to the SDRED database [47], IacE belongs to the HFAM1 family of the classical short-chain dehydrogenases/reductases. All major structural motifs of this family can be identified in the sequence of IacE (Supplementary Figure S21): four active site residues (Asn111, Ser139, Tyr153, and Lys157), NNAG motif, stabilizing the central β-sheet (Asn96, Asn97, Ala98, Gly99), a glycine-rich motif for the binding of NAD(P)H (9ThrGlyAlaAlaArgGlyLeuGly16) and PG motif (Pro193, Gly194). IacE also clusters with other NAD(P)^+^-dependent short chain dehydrogenases/reductases in a phylogenetic tree (Supplementary Figure S23). Thus, it is unlikely that IacE uses H_2_O directly to produce DOAA. A more plausible mechanism would be the spontaneous addition of water to Ox-IAA forming a diol derivative, which could then be oxidized by IacE to produce a stable DOAA (Figure 7).

A further conversion of DOAA in *C. glathei* cells might be related to indole catabolism where the conversion of 3-hydroxyindolin-2-one—a structural homolog of DOAA—is performed by IifA, a putative cofactor-independent oxygenase, composed of two domains [41]. Interestingly, both IacI from the proposed IAA degradation pathway in *C. glathei* and the C-terminal domain of IifA contain a SnoaL-fold domain. Therefore, it was hypothesized that IacI, possibly together with IacB, could perform the consecutive conversion of DOAA. However, neither bioconversion with recombinant *E. coli* cells co-expressing IacBI (Supplementary Figure S16) nor in vitro conversion with soluble fractions of *E. coli* lysates containing IacB and IacI (Supplementary Figure S17) resulted in the transformation of DOAA. One explanation might be that *E. coli* is not a suitable host for obtaining active Iac proteins other than IacAE, since all biotransformation reactions of DOAA were achieved with recombinant producers closely related to the original IAA-degrading organism [23,24,28]. So far, the metabolic gap between DOAA and catechol during IAA biodegradation as well as functions of other Iac proteins remains unfilled.

### 4.3. Perspectives of the Bioconversion of IAA Homologs

The growth of IAA-degrading microorganisms, namely *P. putida* 1290, has been tested on IAA derivatives (IPA, IBA, indole-3-acetaldehyde, indole-3-acrycil acid, indole-3-lactic acid, indole-3-pyruvic acid, naphthalene acetic acid) but no growth was observed, except for indole-3-acetaldehyde [21]. In spite of that, this report describes the conversion of some of these compounds to DOAA-like derivatives by using an *E. coli*-based bioconversion platform carrying IacAE protein combination. This implies that either IacAE proteins from different organisms have different substrate scope, or other Iac proteins of the degradation pathway do not accept DOAA-like derivatives as substrates thus preventing the complete assimilation of IAA homologs for growth. Furthermore, IacA was found to be able to oxidize eight out of 11 tested IAA derivatives, while IacE further converted 6 out of 8 Ox-IAA-like derivatives into DOAA homologs, suggesting a wide substrate scope, which could only be limited by the availability of substrates. It should be stressed that 2,3-dihydroxyindoline-3-acetic acid (Figure 3 and Figure 7) has two chiral centers at C2 and C3, hence this compound can exist in various enantiomeric and diastereomeric forms. To determine which one is a true substrate of IacE and which is an absolute stereostructure of the DOAA, additional studies have to be carried out.

Both IPA and IBA are present in different environments. Yet, no organisms or enzymes with IPA- or IBA-degradation capability have been reported. By showing that the IacAE enzyme system can convert IPA and IBA to corresponding DOAA-like derivatives, this report suggests that IacAE could be a central part of such hypothetical pathway. From the practical point of view, these enzymes are attractive for the engineering of the artificial metabolic cascades converting 3-substituted carboxy derivatives of indole. Such products could be of special importance since *N*-heterocyclic compounds with DOAA scaffold exhibit important biological activities. For example, convolutamydines (4,6-dibromo-3-hydroxyoxindoles), specifically a convolutamydine A, promoted the development of normal cell characteristics in a tumor cell line HL-60 [48]. DOAA is also a building block of plethora of biologically-active compounds: proteasome inhibitor TMC-95 [49], inhibitors of tubulin polymerization celogentins [50], medicinal plant-derived alkaloids paratunamides [51], and arundaphine [52]. Also, as IPA was shown to possess antitubercular activity [18], it could represent a perfect scaffold for a lead compound during the target-based drug optimization [53] and IacA or IacAE systems could be useful for the development of such compounds with improved antitubercular activity. Some of these compounds have been chemically synthesized, usually via enol reactions [54], which often require the use of organic solvents, acids, and extreme conditions. Enzymatic synthesis of DOAA derivatives by using IacAE offers several advantages, including mild reaction conditions and, probably, enantioselectivity.

## 5. Conclusions

In this report, the ability of *Caballeronia glathei* strain DSM50014 to use indole-3-acetic acid as a sole carbon and energy source was demonstrated. The first enzyme in this process, a flavin-dependent oxygenase IacA was shown to convert IAA into 2-hydroxyindole-3-acetic acid without the incorporation of oxygen from H_2_^18^O, supporting the role of IacA as a hydroxylase rather than an epoxidase. The conversion of Ox-IAA into the central intermediate 3-hydroxy-2-oxindole-3-acetic acid was shown to be catalyzed by IacE and without the requirement for IacB. Furthermore, the oxygen atom incorporated into DOAA was derived from water. Also, IacA and IacE were shown to convert a wide range of indole derivatives, including biologically active compounds indole-3-propionic acid and indole-3-butyric acid, into corresponding DOAA-like derivatives.

## Figures and Tables

**Table 1:** Substrate scope of IacA and IacE. Products of the IacA-catalyzed reaction—homologs of Ox-IAA, products of IacE-catalyzed reaction—homologs of DOAA. “+” indicates successful conversion, “−“ indicates no conversion, NA—not analyzed. Bioconversion efficiency of IacA is presented as mean ± SD.

Substrate	Structure	Activity	
		IacA (Bioconversion Efficiency, %)	IacE
**IAA**	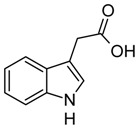	+ (34 ± 8)	+
**IPA**	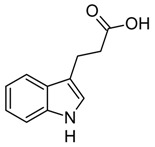	+ (22 ± 5)	+
**IBA**	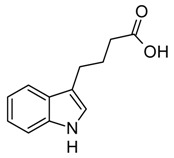	+ (21 ± 5)	+
**Indole**	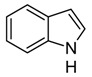	+ (NA)	−
**3-(2-hydroxyethyl)indole**	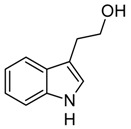	+ (32 ± 7)	+
**Ethyl-3-indole-acetate**	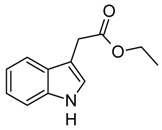	+ (19 ± 6)	+
**3-(3-hydroxypropyl)indole**	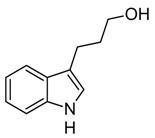	+ (37 ± 8)	+
**3-indoleacetonitrile**	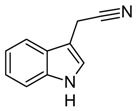	+ (>90)	+
**Indole-3-acetamide**	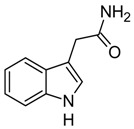	+ (28 ± 5)	−
**Indole-3-lactic acid**	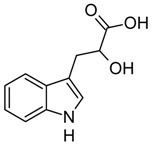	+ (43 ± 11)	−
**Tryptamine**	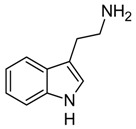	−	NA
**Indole-3-acrycil acid**	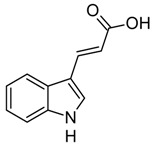	−	NA
**Indole-3-carboxylic acid**	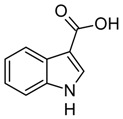	−	NA

## Supplementary Materials

The following are available online at https://www.mdpi.com/2218-273X/10/4/663/s1, Table S1: Oligonucleotides used in this study, Table S2: Characteristics of bacterial strains used in this study, Figure S1: Growth of *Caballeronia glathei* DSM50014 on M9 minimal medium, Figure S2: SDS-PAGE of IacA and IacE co-expression in *E. coli,* Figure S3: SDS-PAGE of IacB expressed in *E. coli*, Figure S4: SDS-PAGE of IacB and IacI expressed in *E. coli*, Figure S5: HPLC chromatograms of bioconversion products, Figure S6: HPLC chromatograms of bioconversion products, Figure S7: HPLC chromatograms of bioconversion products, Figure S8: HPLC chromatograms of bioconversion products, Figure S9: HPLC chromatograms of bioconversion products, Figure S10: HPLC chromatograms of bioconversion products, Figure S11: HPLC chromatograms of bioconversion products, Figure S12: HPLC chromatograms of bioconversion products, Figure S13: HPLC chromatograms of bioconversion products, Figure S14: HPLC chromatograms of bioconversion products, Figure S15: HPLC chromatograms of bioconversion products, Figure S16: HPLC chromatograms of bioconversion products, Figure S17: HPLC chromatograms of bioconversion products, Figure S18: HPLC chromatograms of bioconversion products, Figure S19: ^13^C NMR spectrum of DOAA, Figure S20: ^1^H NMR spectrum of DOAA, Figure S21: conservative sequence motifs in the sequence of IacE. Figure S22: Phylogenetic tree of IacA. Amino acid sequences of group D flavin-dependent oxygenase were picked according to [55]. Sequences were aligned by using the ClustalW algorithm, a maximum-likelihood tree was constructed by using MEGA7 software [56] with 1000 bootstrap replications. The percentage of replicate trees in which the associated taxa clustered together in the bootstrap test are shown. Figure S23: Phylogenetic tree of IacE. Amino acid sequences of short-chain dehydrogenases/reductases (SDR) were picked from each SDR family according to [57]. Sequences were aligned by using the ClustalW algorithm, a maximum-likelihood tree was constructed by using MEGA7 software [56] with 1000 bootstrap replications.
