# Supplementary material for: Bioconversion of Biologically Active Indole Derivatives with Indole-3-Acetic Acid-Degrading Enzymes from Caballeronia glathei DSM50014
Source: Biomolecules. 2020 Apr 24;10(4):663. doi: 10.3390/biom10040663 (PMC7225977; doi:10.3390/biom10040663)
Supplement: Supplementary file 1 [file biomolecules-10-00663-s001.pdf]

biomolecules

MDPI

Supplementary Material for

Bioconversion of Biologically Active Indole Derivatives with Indole-3-Acetic Acid-Degrading Enzymes from *Caballeronia glathei* DSM50014

Mikas Sadauskas *, Roberta Statkevičiūtė, Justas Vaitekūnas and Rolandas Meškys.

Department of Molecular Microbiology and Biotechnology, Institute of Biochemistry, Life Sciences Center, Vilnius University, Saulėtekio al. 7, Vilnius, LT-10257, Lithuania; roberta.statkeviciute@gmc.stud.vu.lt (R.S.), justas.vaitekunas@bchi.vu.lt (J.V.), rolandas.meskys@bchi.vu.lt (R.M.)

* Correspondence: mikas.sadauskas@bchi.vu.lt; Tel.: +37062230055

Table S1. Oligonucleotides used in this study. Recognition sequences of restriction endonucleases are underlined.

Oligonucleotide	Oligonucleotide Sequence
glathei-iacA-Nde-F	5'-CATATGTCCGCAACCGCCCTCGC-3'
glathei-iacA-Xho-R	5'-CTCGAGTTAGAGATAGCCCGGAATCG-3'
iacE-Nde-F	5'-CGCATATGTGCTGCACAGCATC-3'
iacE-Xho-R	5'-CTCGAGTCAGCGCATGTAGAGGC-3'
iacB2-Nde-F	5'-CAACATATGAGCCAGACAACAACCTTACG-3'
iacB2-Xho-R	5'-CTCGAGTTACTGAATAATCAGTTCTCGAC-3'

Table S2. Characteristics of bacterial strains used in this study.

Strain	Characteristics	Source
Escherichia coli DH5α	Host for cloning and plasmid isolation	Novagen, Germany
Escherichia coli BL21 (DE3)	Host for protein expression and bioconversion	Novagen, Germany
Caballeronia glathei DSM50014	Degrader of indole-3-acetic acid	DSMZ, Germany
Acinetobacter sp. O153	Degrader of indole	[41]

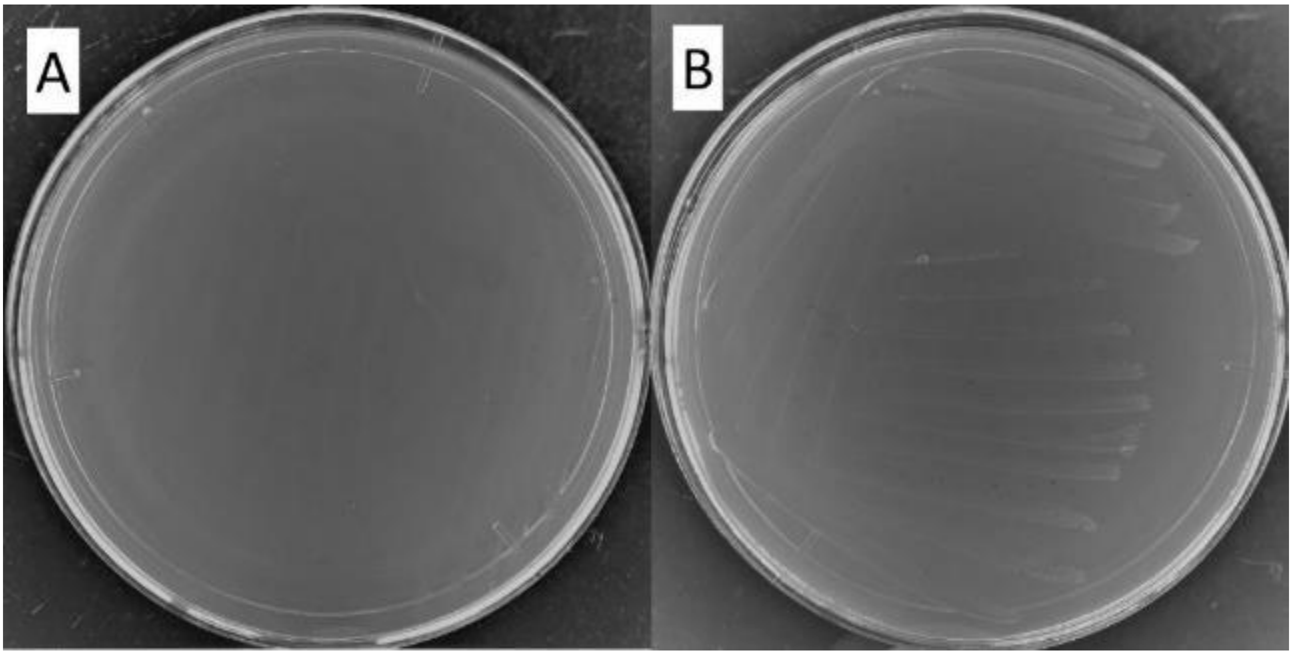

Figure S1. Growth of *Caballeronia glathei* DSM50014 on M9 minimal medium (**A**) and M9 medium supplemented with 1 mM IAA (**B**).

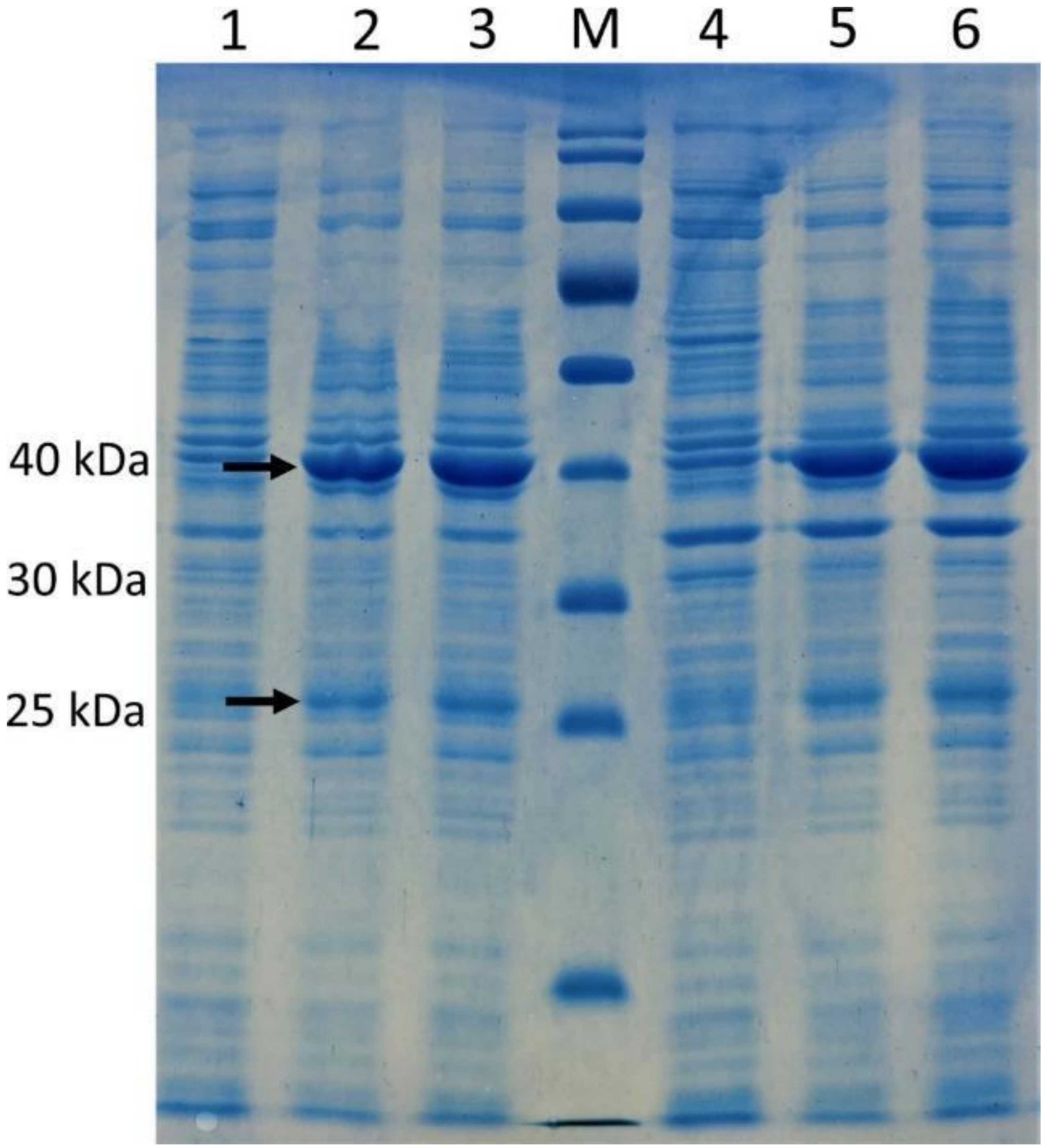

Figure S2. SDS-PAGE of IacA and IacE co-expression in *E. coli*. 1—soluble fraction of *E. coli* BL21 (DE3) cells with no plasmid , 2—cell lysate of *E. coli* BL21 (DE3) cells incubated at 16 °C and co-expressing IacA and IacE, 3—soluble fraction of *E. coli* BL21 (DE3) cells incubated at 30 °C and co-expressing IacA and IacE, 4—soluble fraction of *E. coli* BL21 (DE3) cells with no plasmid, 5—soluble fraction of *E. coli* BL21 (DE3) cells incubated at 16 °C and co-expressing IacA and IacE, 6—soluble fraction of *E. coli* BL21 (DE3) cells incubated at 30 °C and co-expressing IacA and IacE, M—PageRuler Prestained Protein Ladder (ThermoFisher Scientific). Arrows indicate the bands of the IacA and IacE proteins.

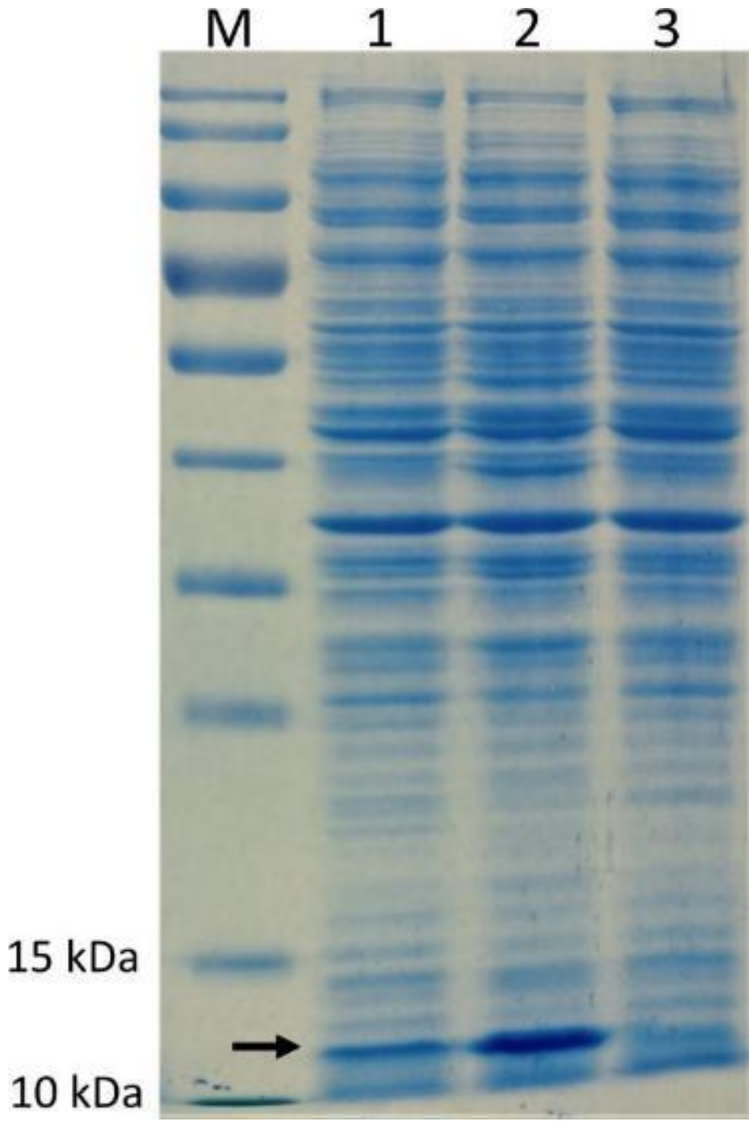

Figure S3. SDS-PAGE of IacB expressed in *E. coli*. 1—soluble fraction of *E. coli* BL21 (DE3) cells incubated at 16 °C and expressing IacB , 2—soluble fraction of *E. coli* BL21 (DE3) cells incubated at 30 °C and expressing IacB, 3—soluble fraction of *E. coli* BL21 (DE3) cells with no plasmid, M—PageRuler Prestained Protein Ladder (ThermoFisher Scientific). Arrow indicates the bands of IacB protein.

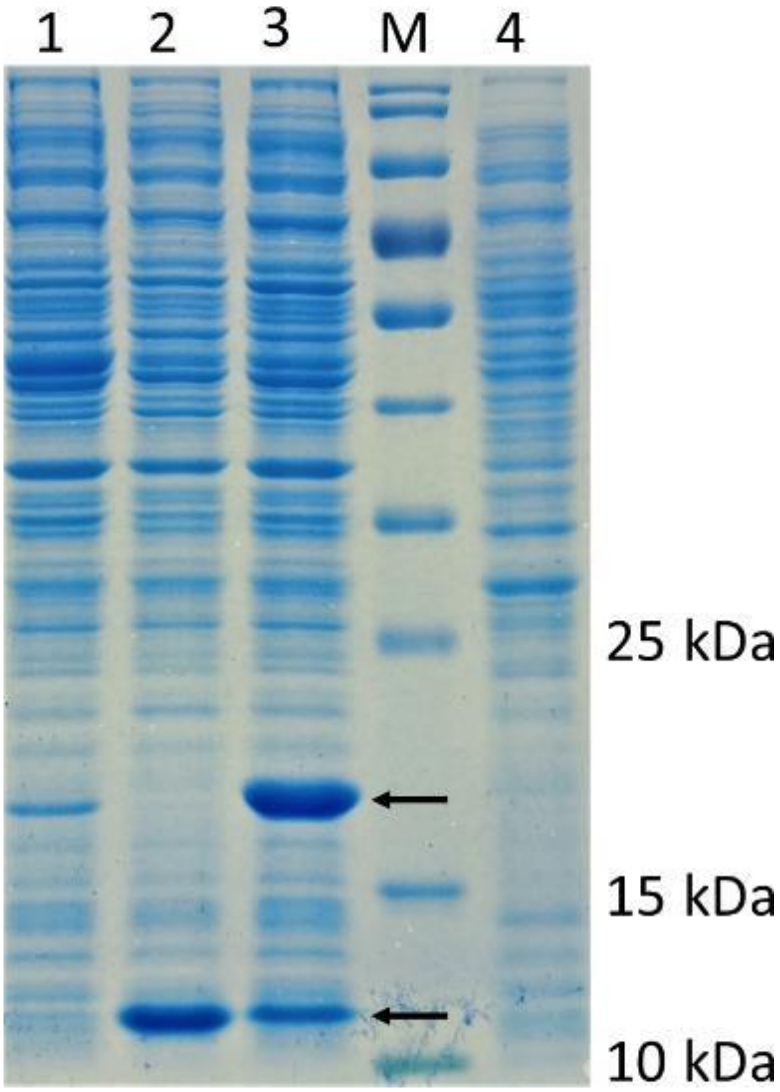

Figure S4. SDS-PAGE of IacB and IacI expressed in *E. coli*. 1 – soluble fraction of *E. coli* BL21 (DE3) cells expressing IacI, 2—soluble fraction of *E. coli* BL21 (DE3) cells expressing IacB, 3—soluble fraction of *E. coli* BL21 (DE3) co-expressing IacB and IacI, 4—soluble fraction of *E. coli* BL21 (DE3) cells with no plasmid, M—PageRuler Prestained Protein Ladder (ThermoFisher Scientific). Arrows indicate the bands of IacB and IacI proteins.

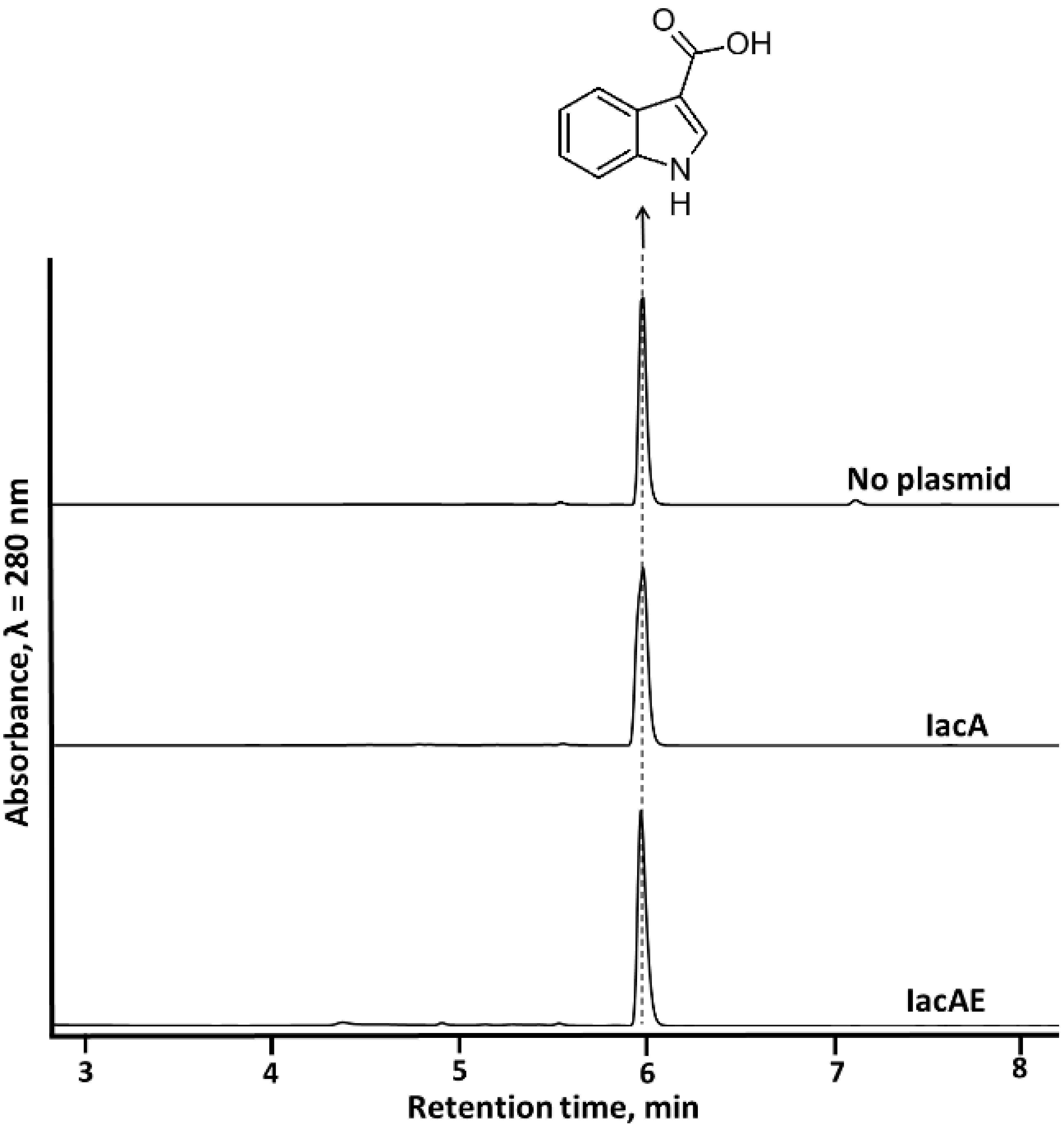

Figure S5. HPLC chromatograms of bioconversion products obtained by using *E. coli* cells expressing different combinations of IacA and IacE proteins and indole-3-carboxylic acid as substrate.

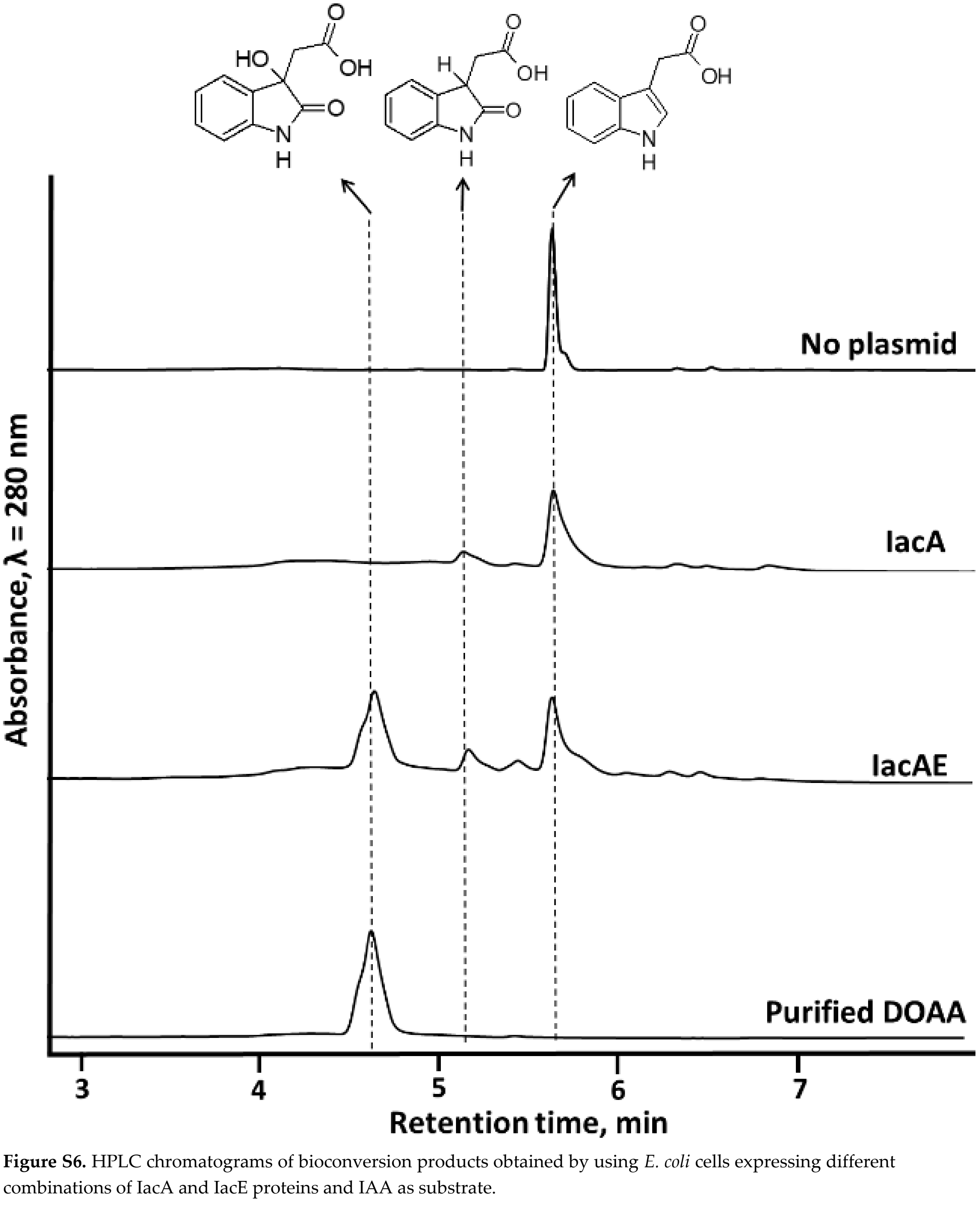

Figure S6. HPLC chromatograms of bioconversion products obtained by using *E. coli* cells expressing different combinations of IacA and IacE proteins and IAA as substrate.

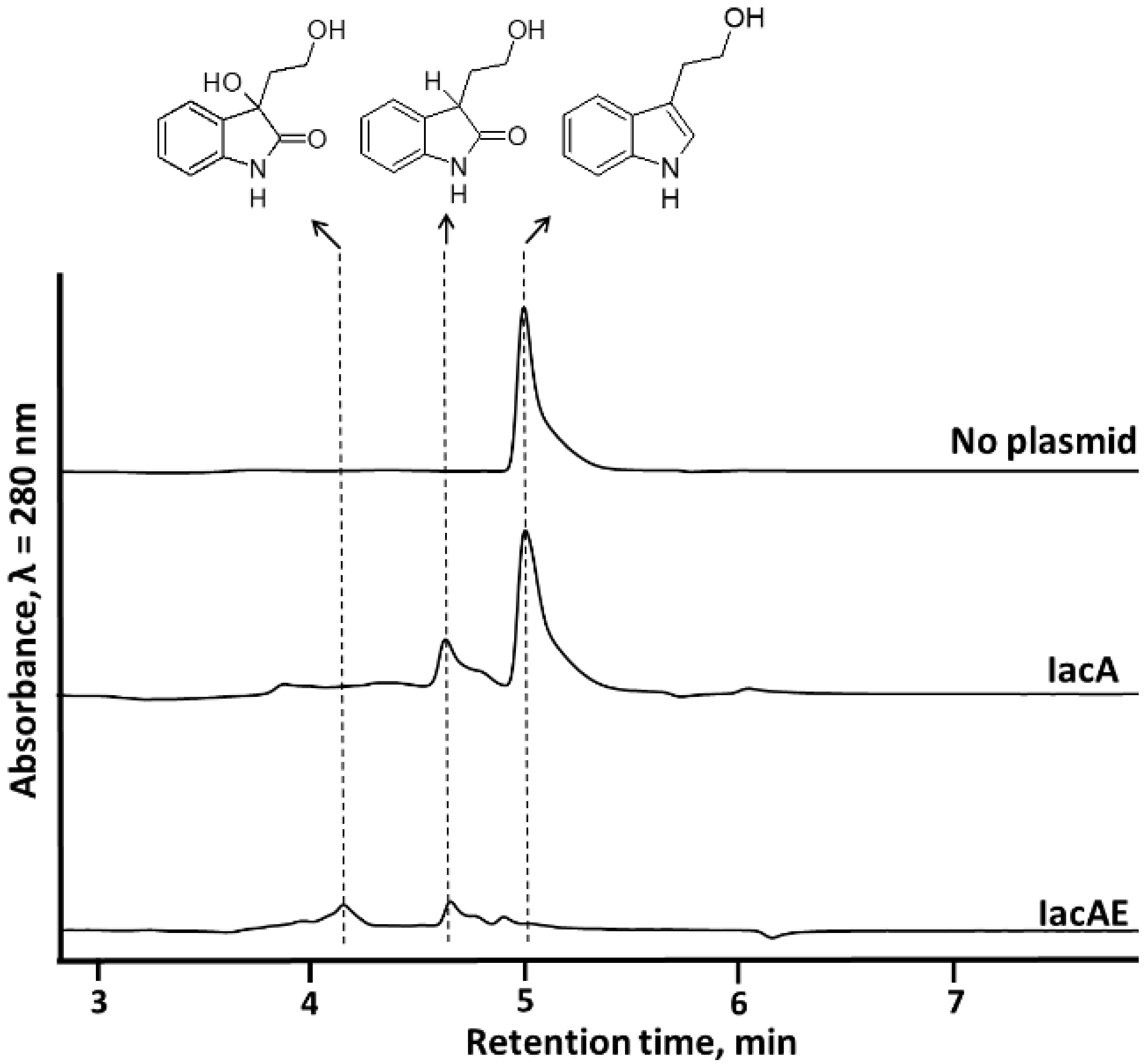

Figure S7. HPLC chromatograms of bioconversion products obtained by using *E. coli* cells expressing different combinations of IacA and IacE proteins and 3-(2-hydroxyethyl)indole as substrate.

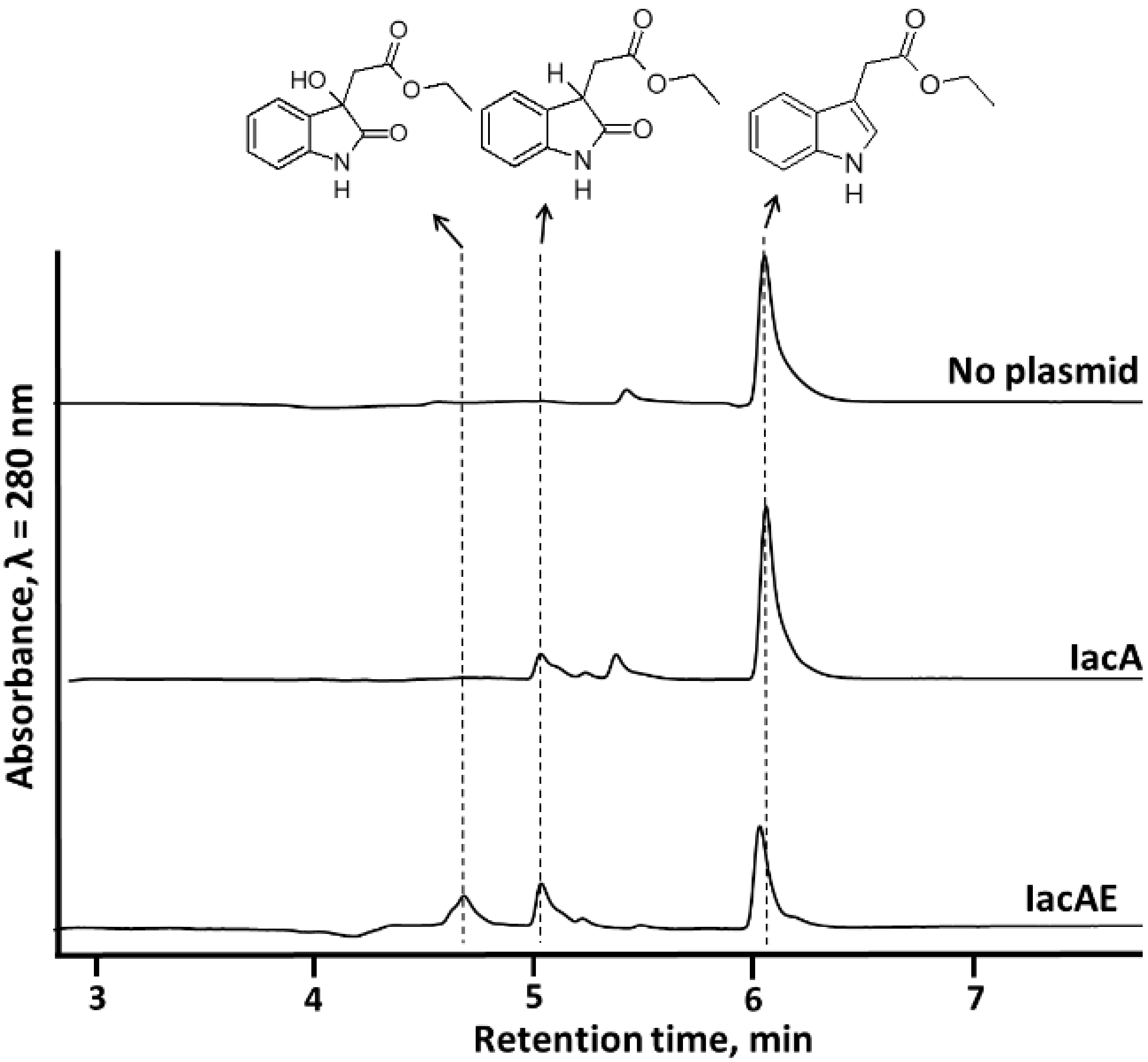

Figure S8. HPLC chromatograms of bioconversion products obtained by using *E. coli* cells expressing different combinations of IacA and IacE proteins and ethyl-3-indole-acetate as substrate.

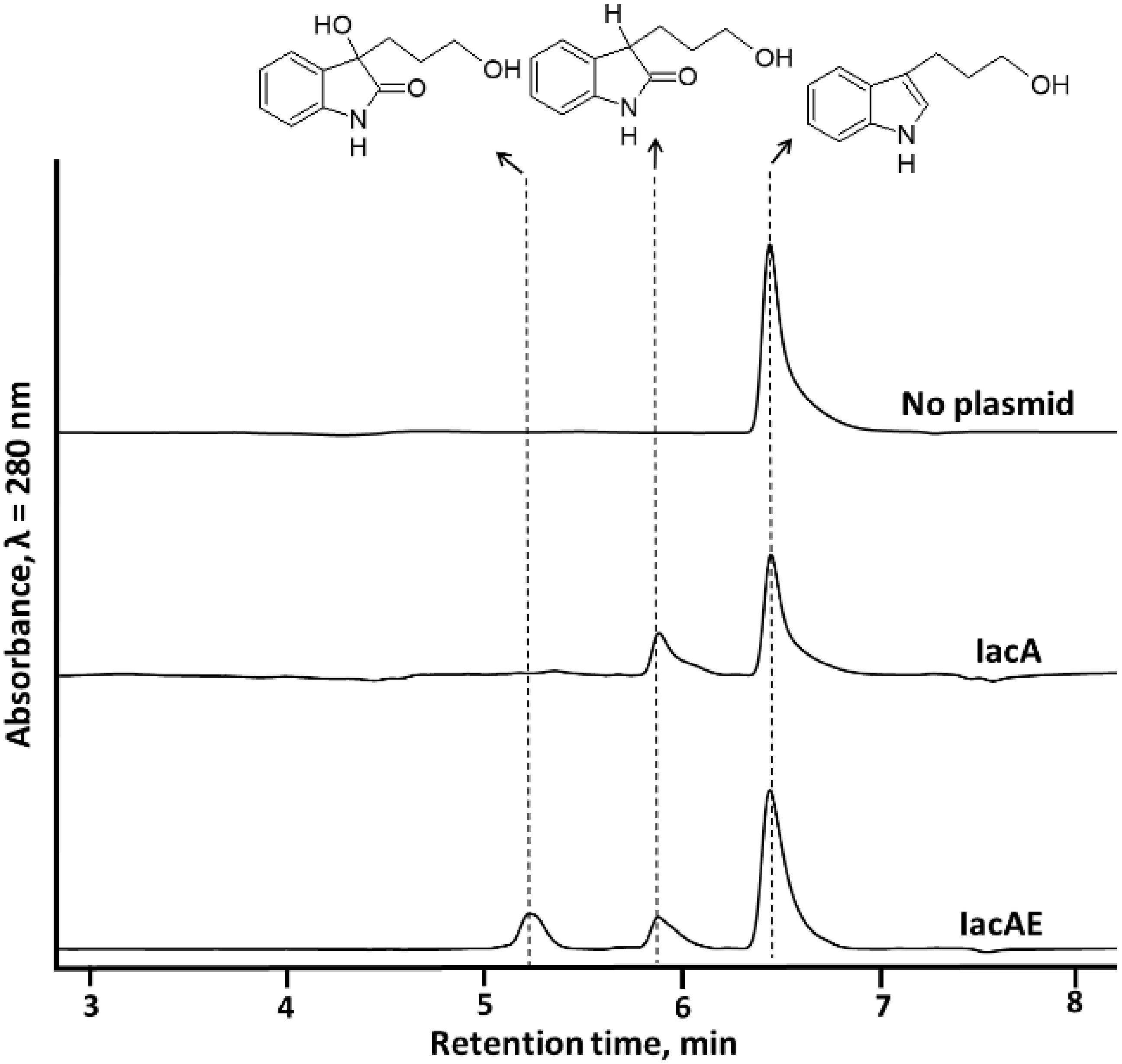

Figure S9. HPLC chromatograms of bioconversion products obtained by using *E. coli* cells expressing different combinations of IacA and IacE proteins and 3-(3-hydroxypropyl)indole as substrate.

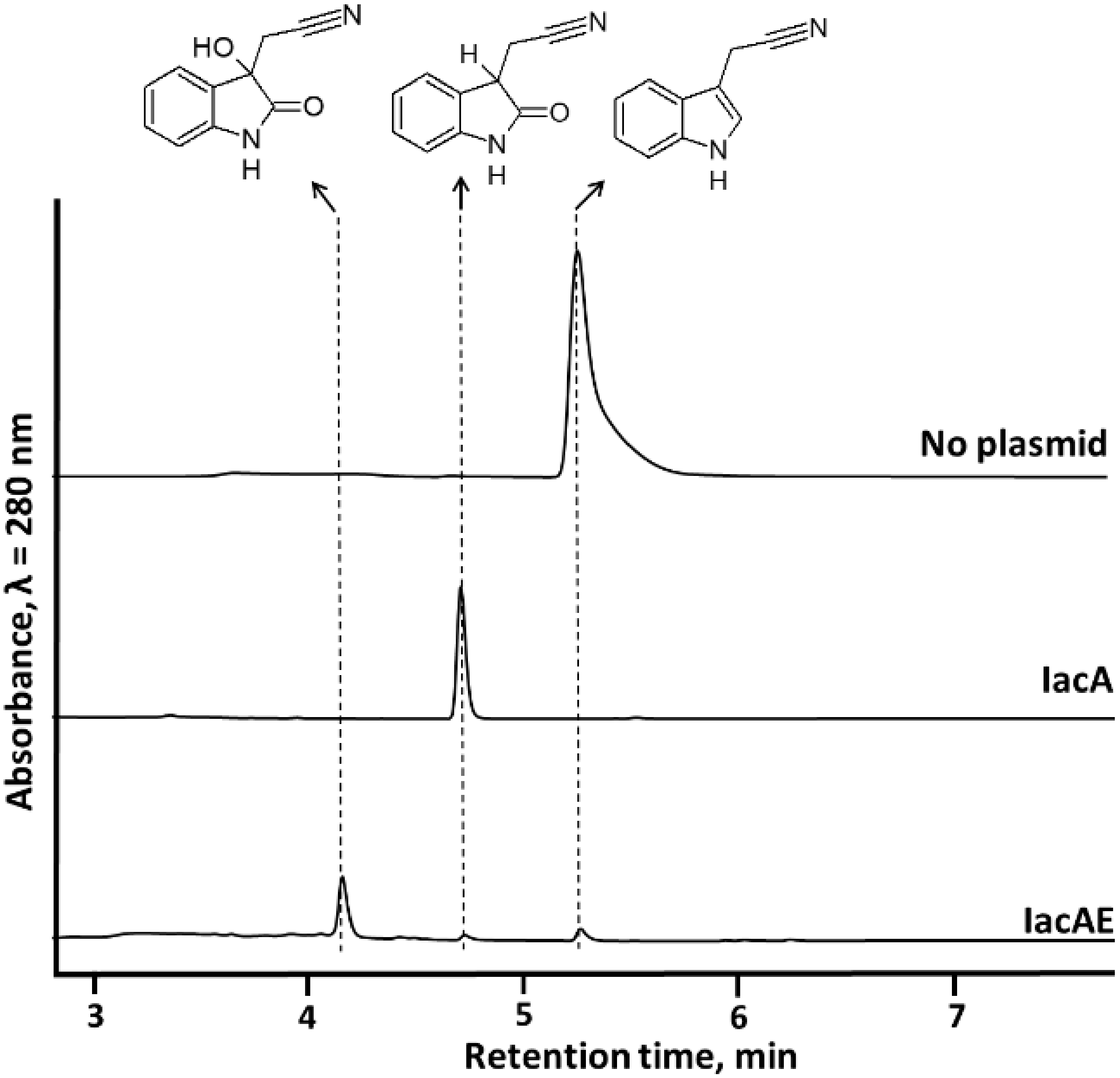

Figure S10. HPLC chromatograms of bioconversion products obtained by using *E. coli* cells expressing different combinations of IacA and IacE proteins and 3-indoleacetonitrile as substrate.

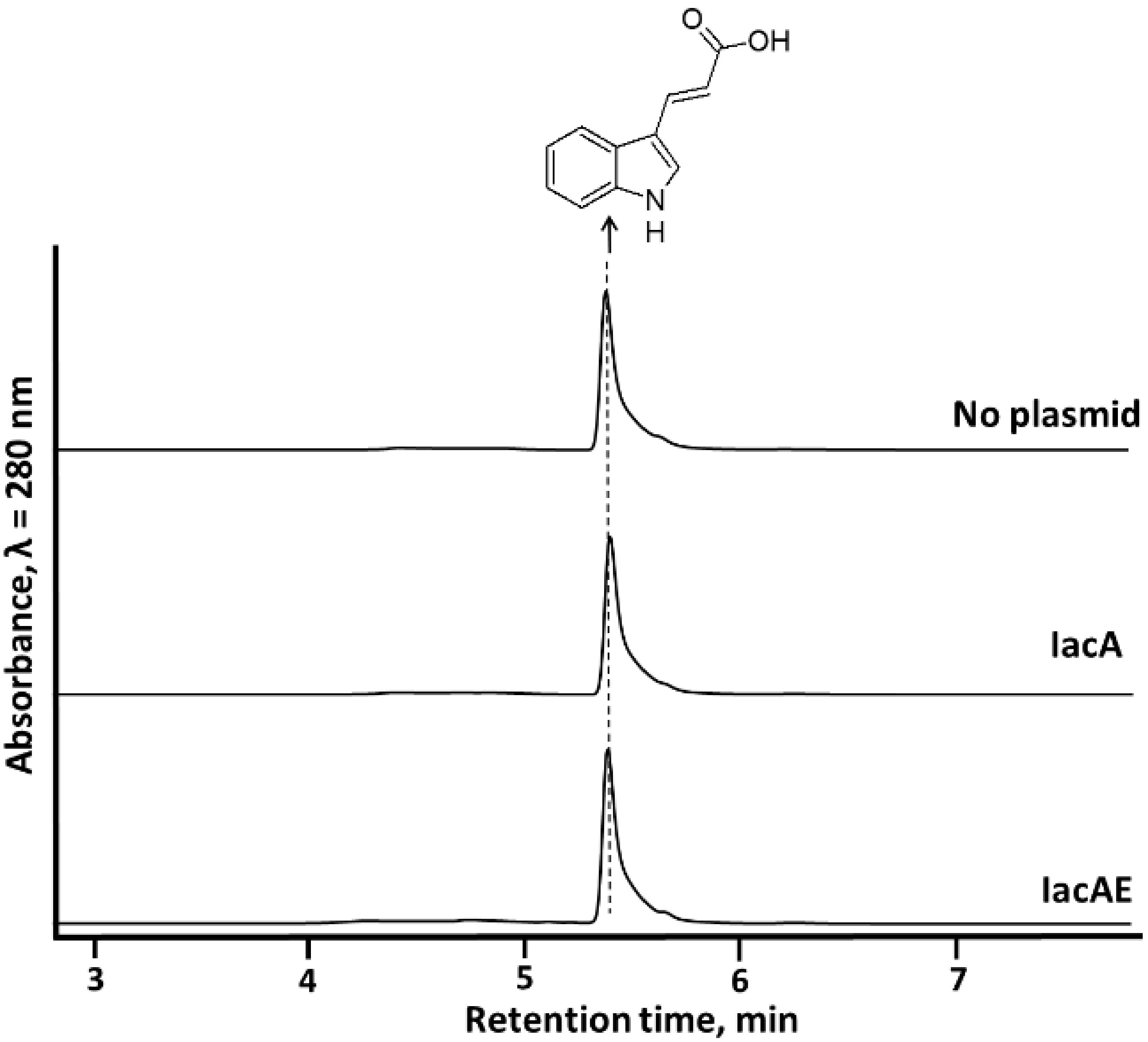

Figure S11. HPLC chromatograms of bioconversion products obtained by using *E. coli* cells expressing different combinations of IacA and IacE proteins and 3-indoleacrycil acid as substrate.

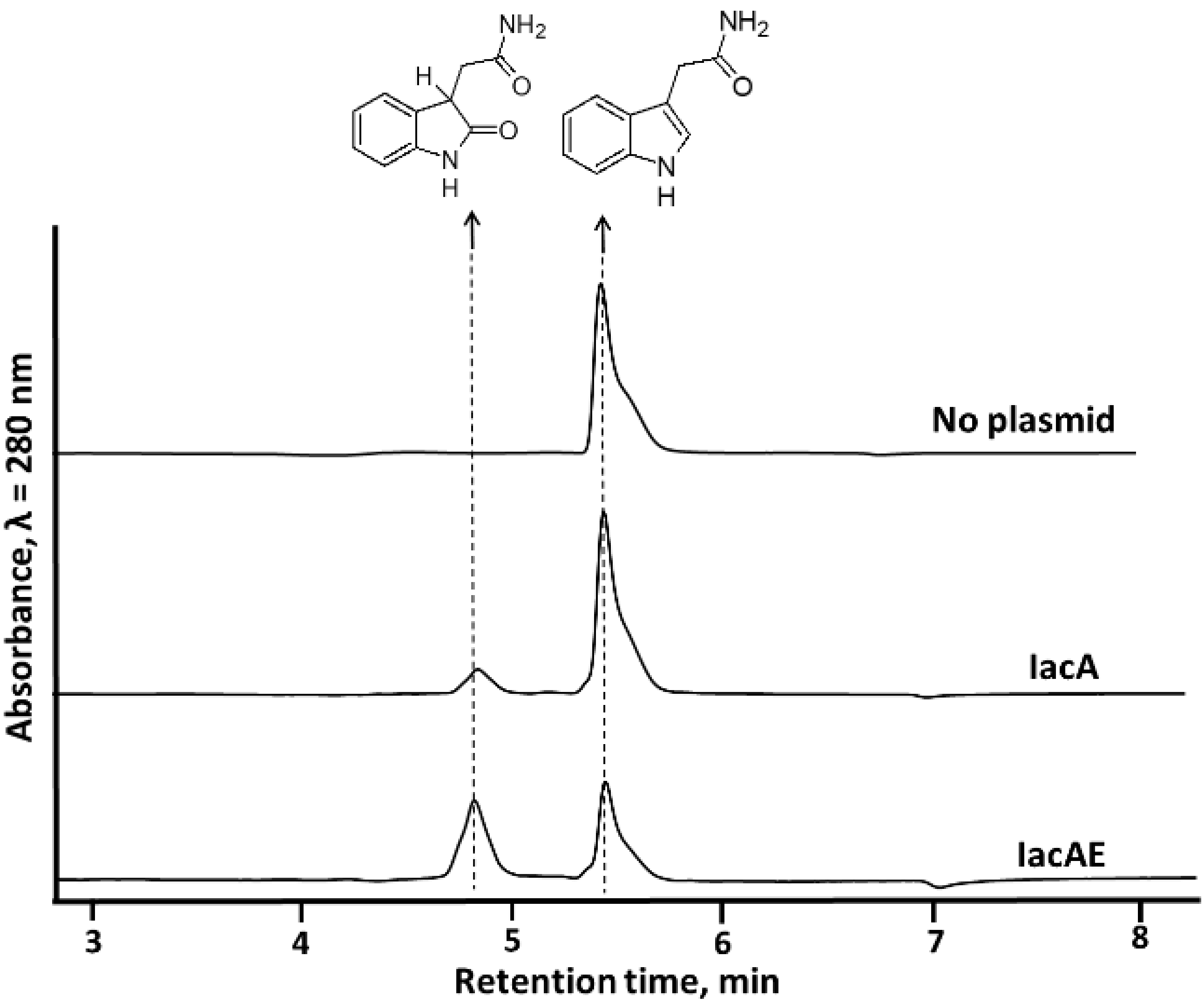

Figure S12. HPLC chromatograms of bioconversion products obtained by using *E. coli* cells expressing different combinations of IacA and IacE proteins and indole-3-acetamide as substrate.

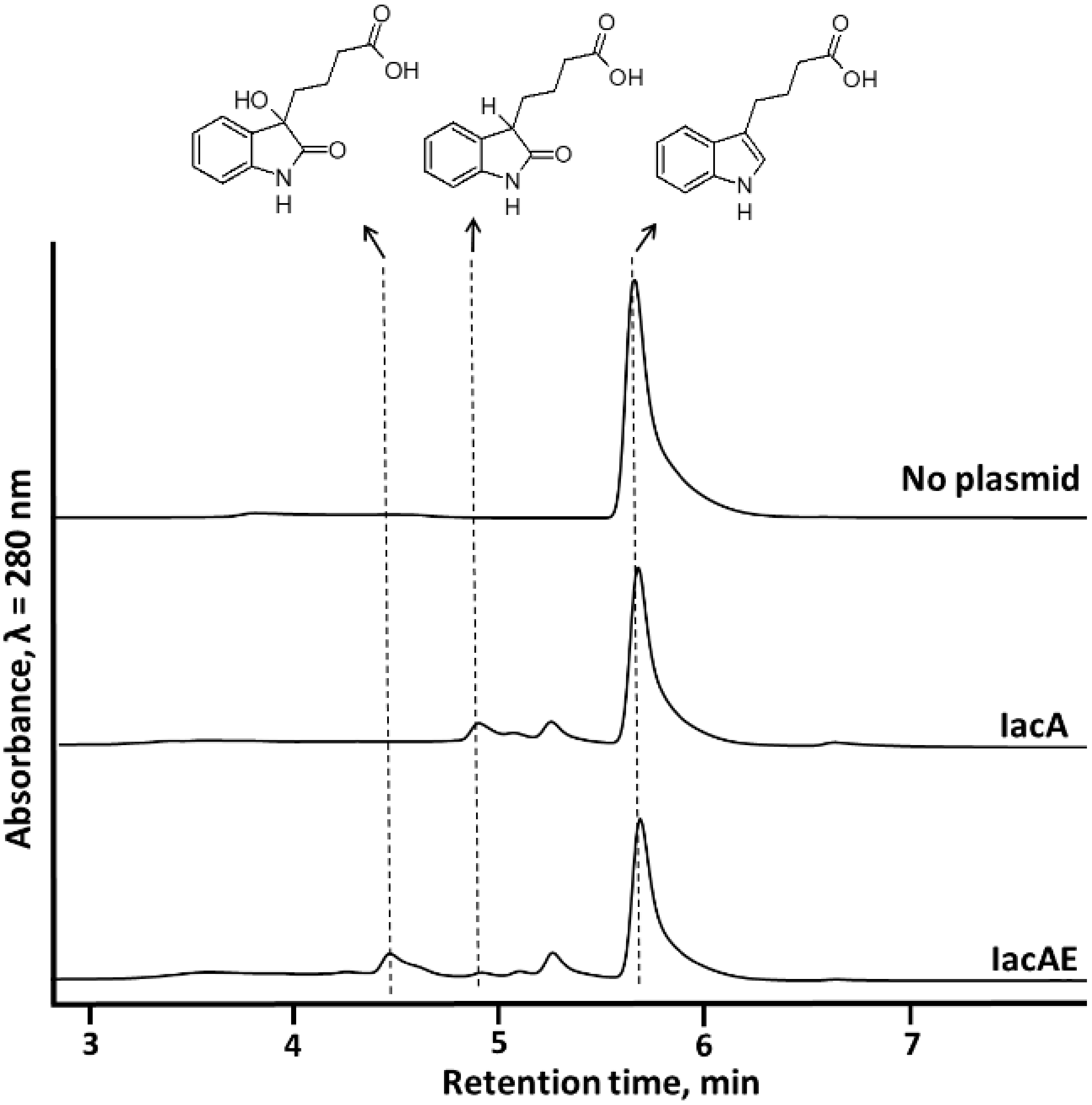

Figure S13. HPLC chromatograms of bioconversion products obtained by using *E. coli* cells expressing different combinations of IacA and IacE proteins and IBA as substrate.

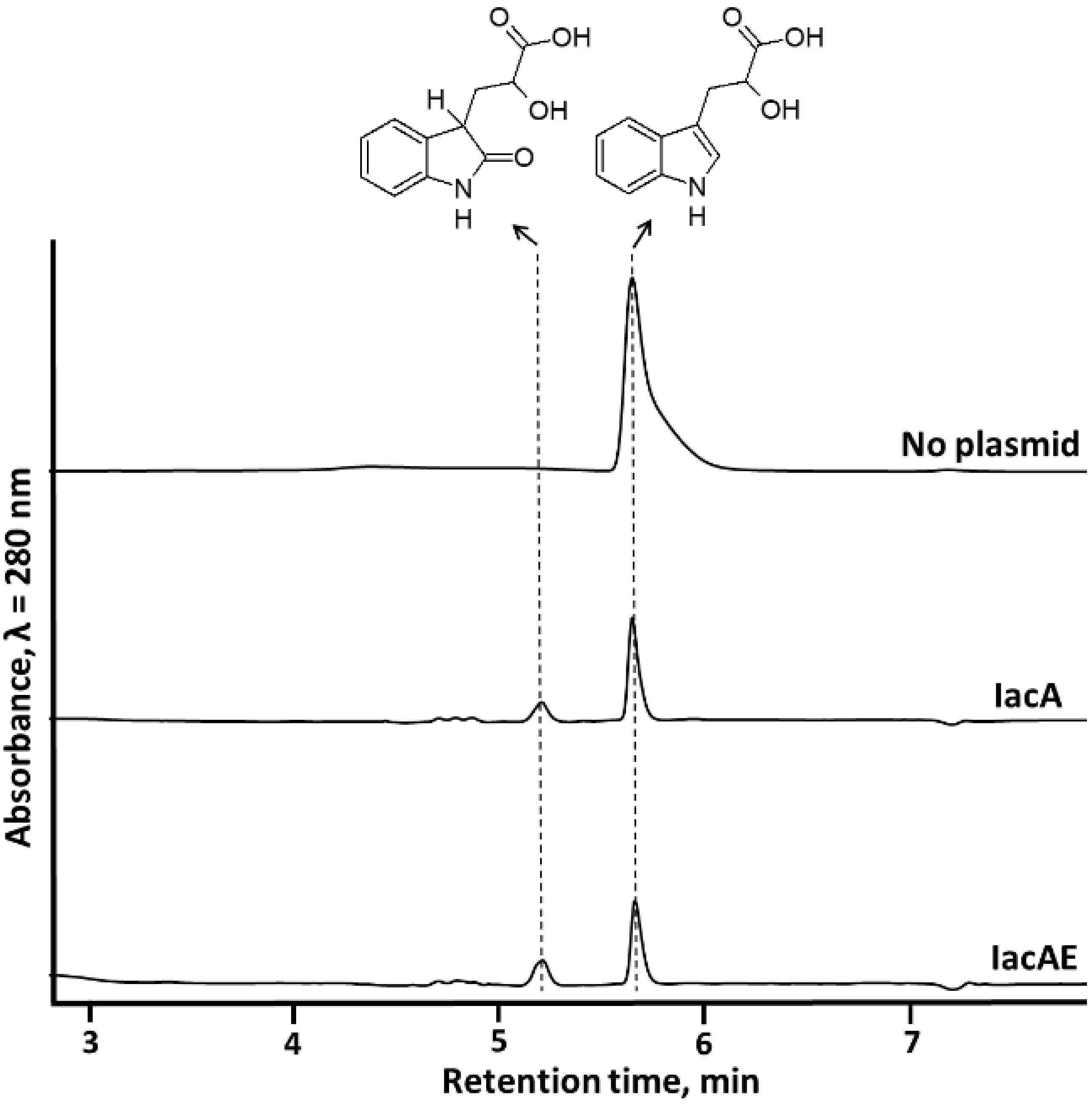

Figure S14. HPLC chromatograms of bioconversion products obtained by using *E. coli* cells expressing different combinations of IacA and IacE proteins and indole-3-lactic acid as substrate.

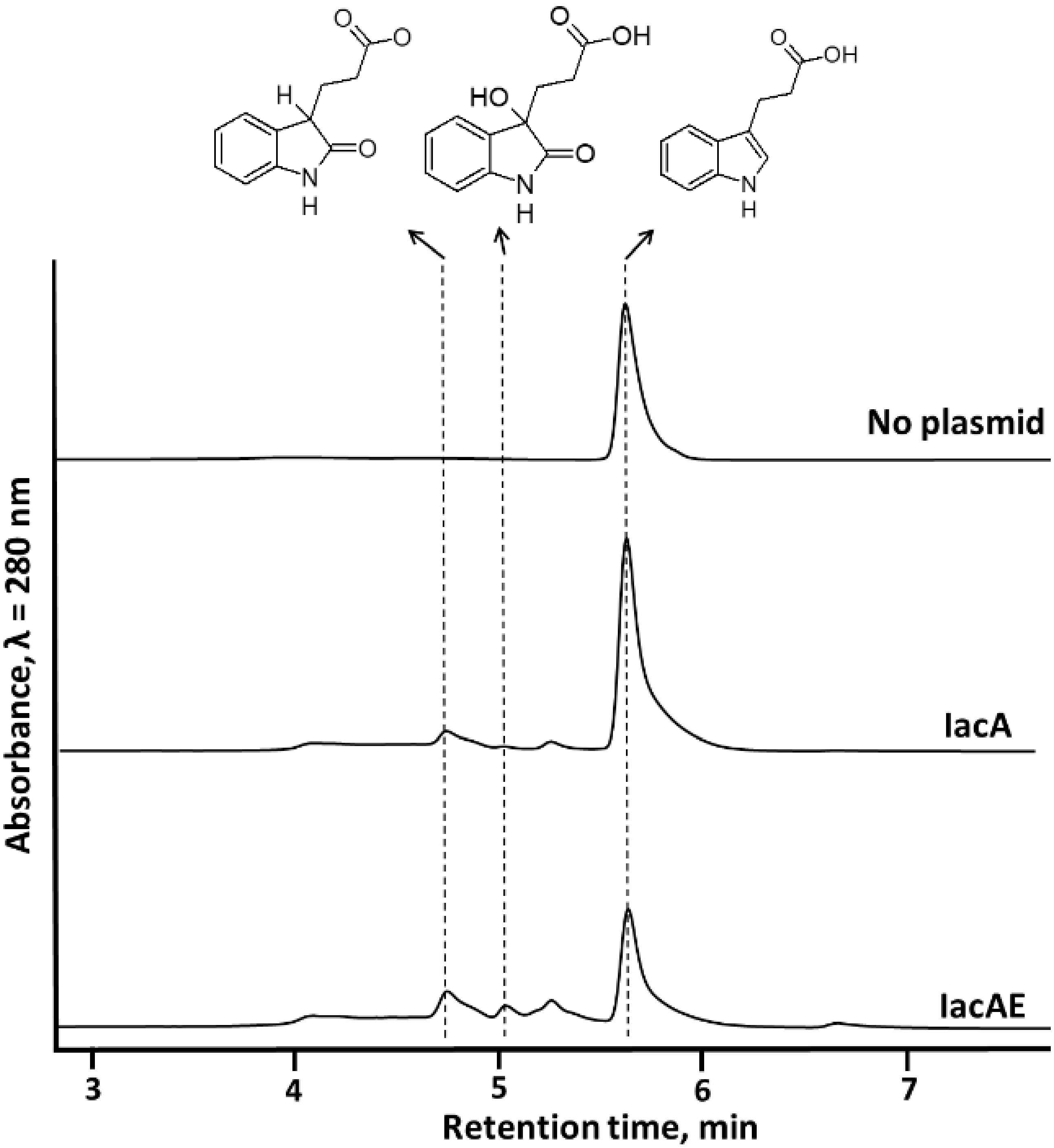

Figure S15. HPLC chromatograms of bioconversion products obtained by using *E. coli* cells expressing different combinations of IacA and IacE proteins and IPA as substrate.

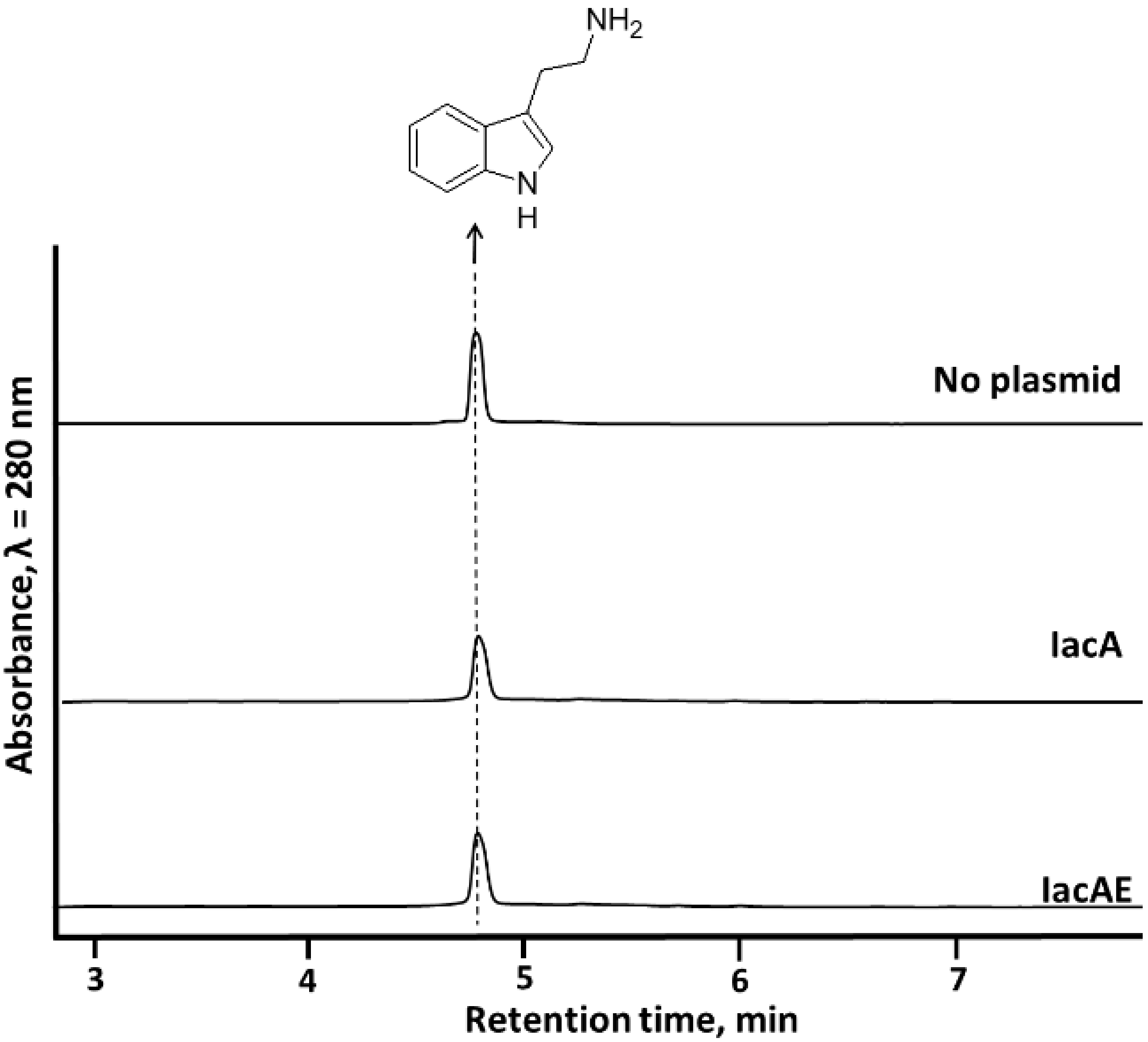

Figure S16. HPLC chromatograms of bioconversion products obtained by using *E. coli* cells expressing different combinations of IacA and IacE proteins and tryptamine as substrate.

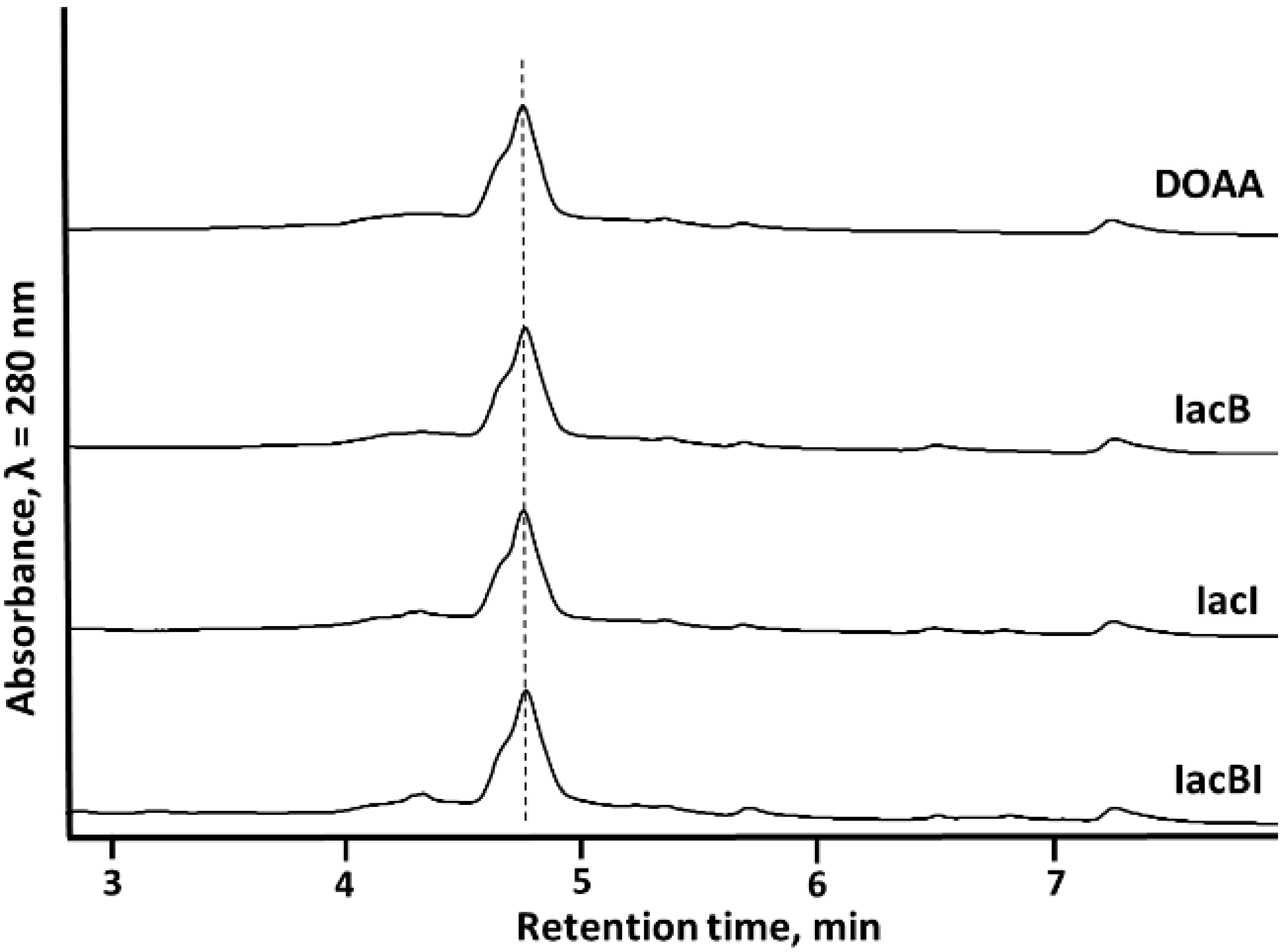

Figure S17. HPLC chromatograms of bioconversion products obtained by using *E. coli* cells expressing different combinations of IacB and IacI proteins and DOAA as substrate.

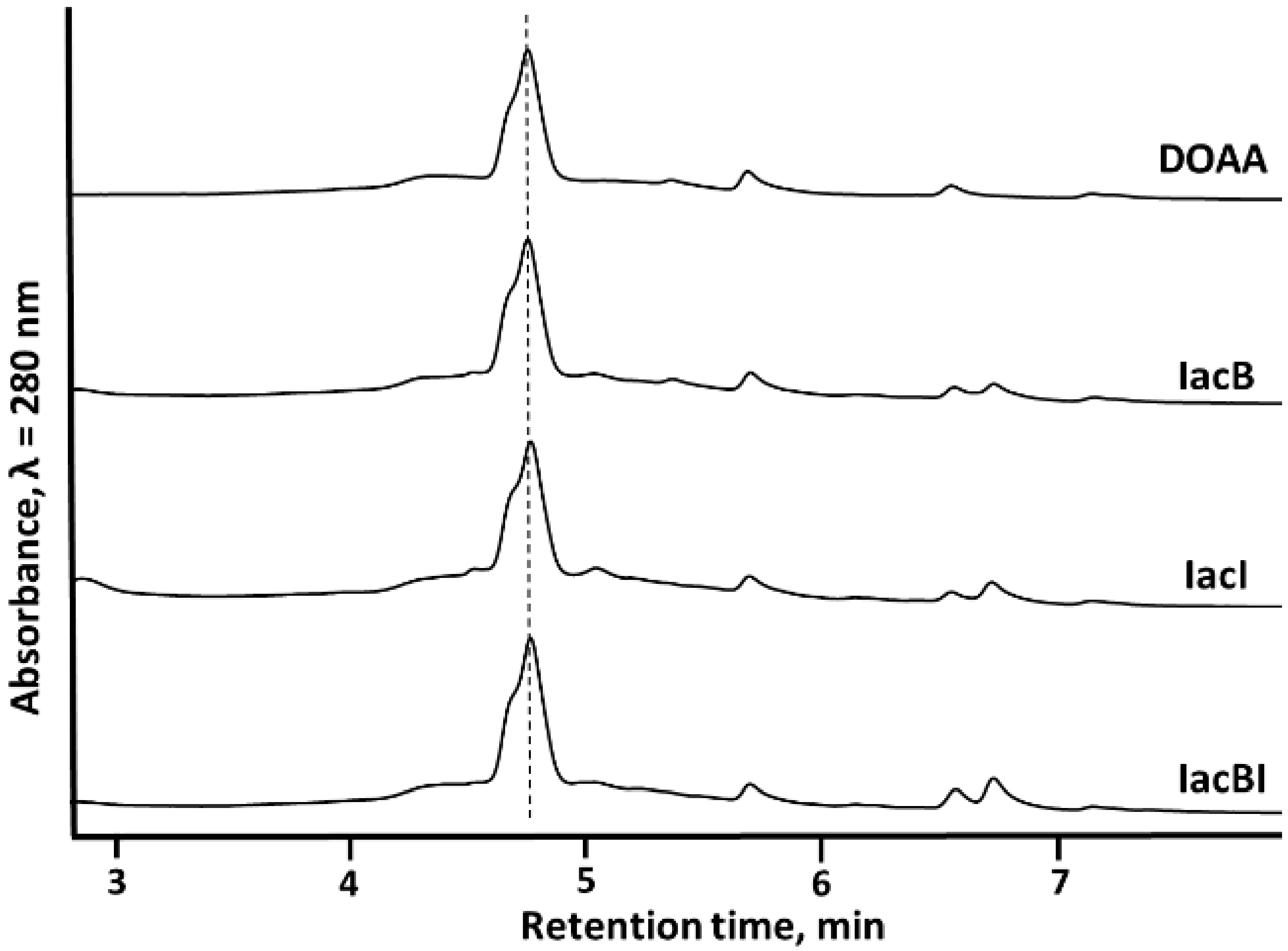

Figure S18. HPLC chromatograms of *in vitro* reaction products obtained by using IacB and IacI proteins and DOAA as substrate.

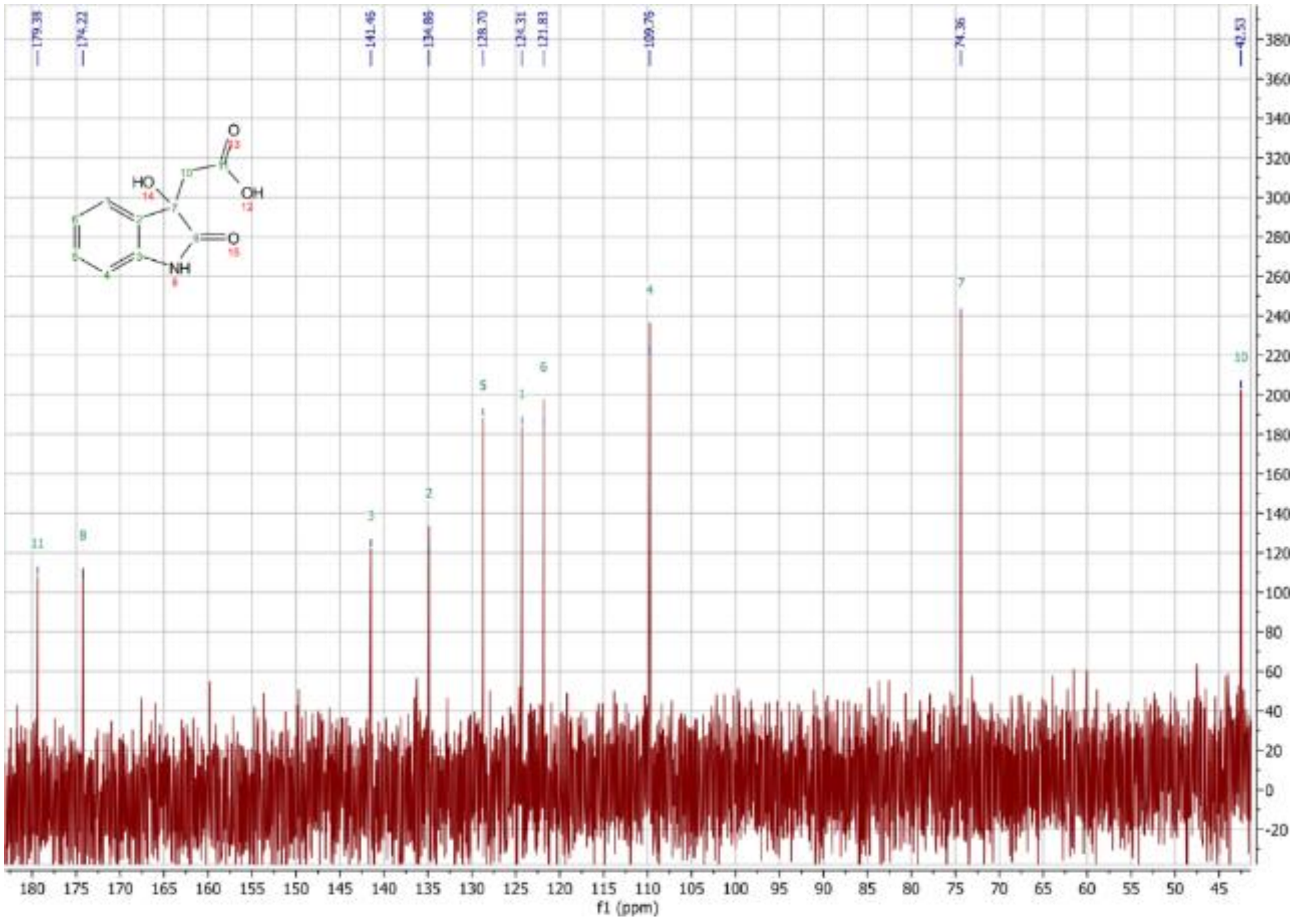

Figure S19. ^{13}C NMR spectrum of DOAA obtained by transforming IAA with *E. coli* cells carrying IacAE proteins.

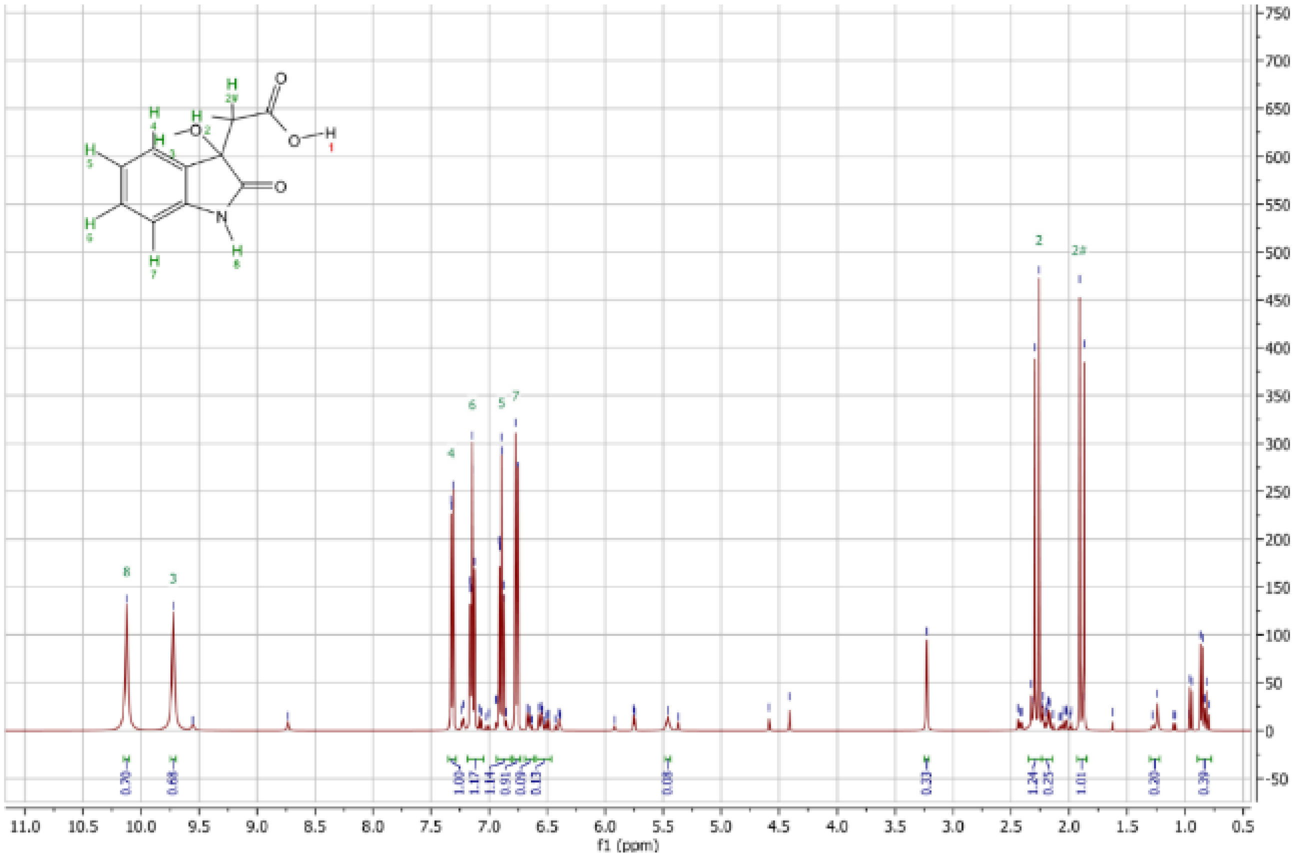

Figure S20. ^{1}H NMR spectrum of DOAA obtained by transforming IAA with *E. coli* cells carrying IacAE proteins.

```
>IacE
MSNRVVLITG    AARGLGAVVA    RRFHEAGYKV    ALADIAVEAA    KTLARELSED    GTSACAIKLD
VSSKADFEAA    RDALLERWDA    IDAIVNNAGA    SKVIPVMEIT    AEQFDQVIDI    NLRSVLFGCQ
VFGQYFAGRG    AGRIVNIASL    AGQNGGSATG    AHYAAAKGGA    ITLTKVFARD    LAPHGVTVNA
ISPGPLDLPI   VHESVPADKL QKVIAGIPVG KLGSAAYIAD VAVLLASADA YFANGACWDV NGGLYMR
```

NAD(P)H binding motif

Conservede alanine

Conserved glycine

Conserved asparagine (binding of nicotinamide)

NNAG motif (Stabilization of beta sheets)

Catalytic tetrad

Catalytic site

PG motif (Direction of reaction)

Figure S21. Conserved sequence motifs in IacE as predicted by SDRED database.

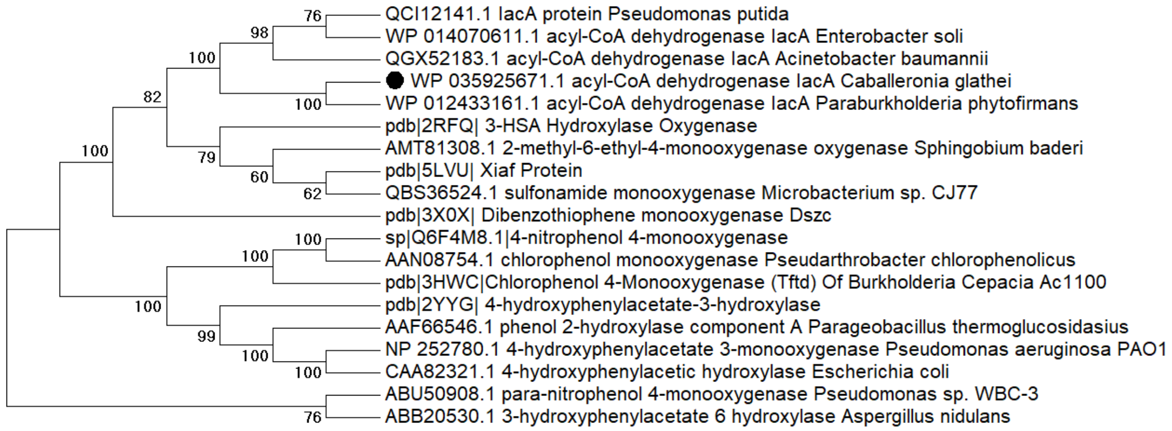

Figure S22. Phylogenetic tree of IacA. Amino acid sequences of group D flavin-dependent oxygenase were picked according to [55]. Sequences were aligned by using the ClustalW algorithm, a maximum-likelihood tree was constructed by using MEGA7 software [56] with 1000 bootstrap replications. The percentage of replicate trees in which the associated taxa clustered together in the bootstrap test are shown.

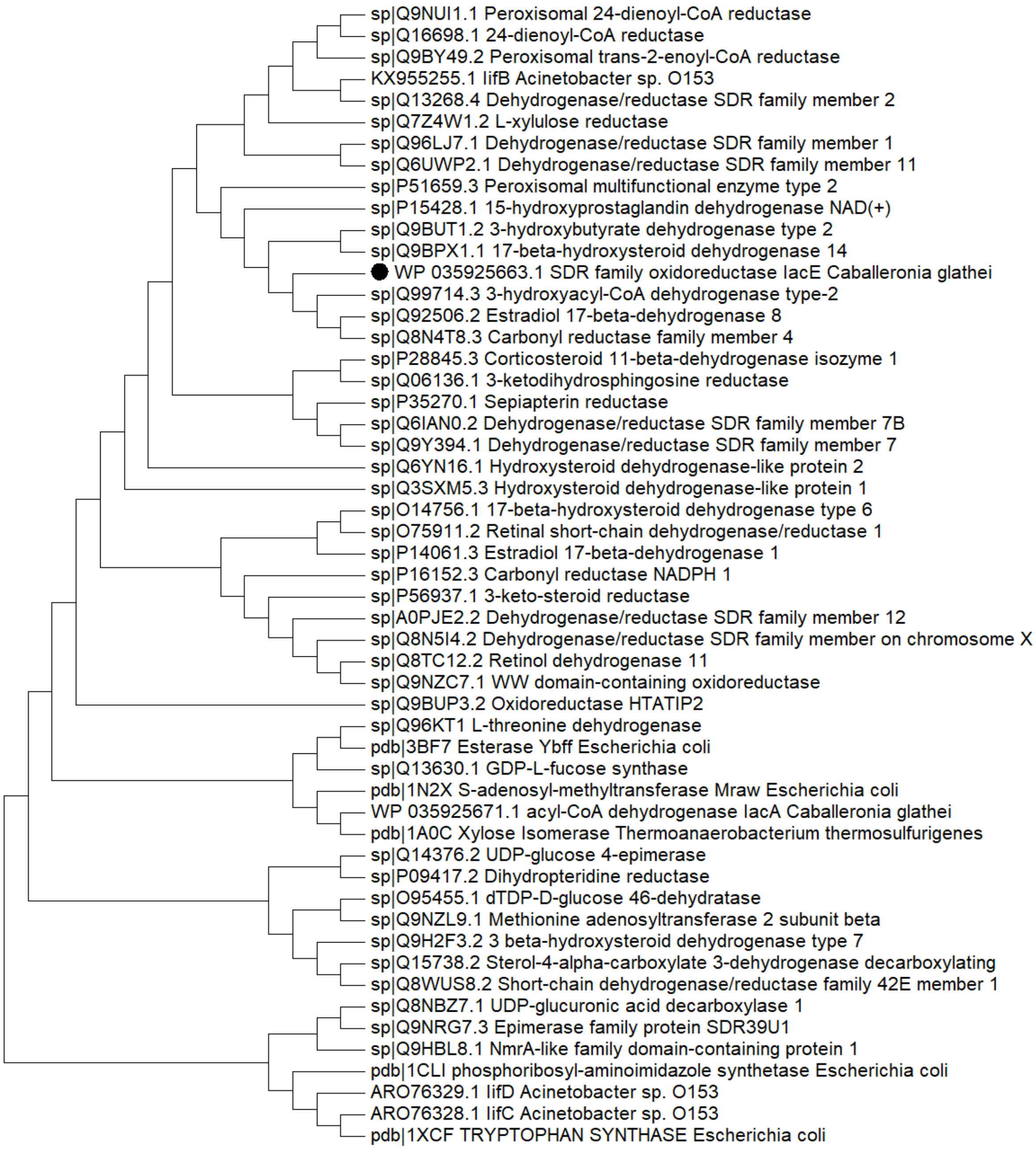

Figure S23. Phylogenetic tree of IacE. Amino acid sequences of short-chain dehydrogenases/reductases (SDR) were picked from each SDR family according to [57]. Sequences were aligned by using the ClustalW algorithm, a maximum-likelihood tree was constructed by using MEGA7 software [56] with 1000 bootstrap replications.
